# Cyclic Peptoid-Peptide Hybrids as Versatile Molecular Transporters

**DOI:** 10.3389/fchem.2021.696957

**Published:** 2021-06-25

**Authors:** Claudine Nicole Herlan, Anna Meschkov, Ute Schepers, Stefan Bräse

**Affiliations:** ^1^ Institute of Biological and Chemical Systems- Functional Molecular Systems (IBCS-FMS), Karlsruhe Institute of Technology (KIT), Karlsruhe, Germany; ^2^ Institute of Functional Interfaces (IFG), Karlsruhe Institute of Technology (KIT), Karlsruhe, Germany; ^3^ Karlsruhe Institute of Technology (KIT), EPICUR European University, Karlsruhe, Germany; ^4^ Institute of Organic Chemistry (IOC), Karlsruhe Institute of Technology (KIT), Karlsruhe, Germany

**Keywords:** peptoid, molecular transport, cyclization, peptidomimetic, amide

## Abstract

Addressing intracellular targets is a challenging task that requires potent molecular transporters capable to deliver various cargos. Herein, we report the synthesis of hydrophobic macrocycles composed of both amino acids and peptoid monomers. The cyclic tetramers and hexamers were assembled in a modular approach using solid as well as solution phase techniques. To monitor their intracellular localization, the macrocycles were attached to the fluorophore Rhodamine B. Most molecular transporters were efficiently internalized by HeLa cells and revealed a specific accumulation in mitochondria without the need for cationic charges. The data will serve as a starting point for the design of further cyclic peptoid-peptide hybrids presenting a new class of highly efficient, versatile molecular transporters.

## Introduction

Biological membranes envelop intracellular structures, protect them from harmful substances and enable diverse physiological processes. The unique function of these phospholipid bilayers is crucial for all processes of life but also hampers the intracellular delivery of therapeutics or other cargos. The efficient internalization of a certain compound is a key step for both pharmaceutical and basic research. Thus, molecules that overcome the physiological barrier, so-called molecular transporters, are constantly needed not only to resolve and understand the fascinating processes of life but also to provide treatments poor in side effects.

**FIGURE 1:**
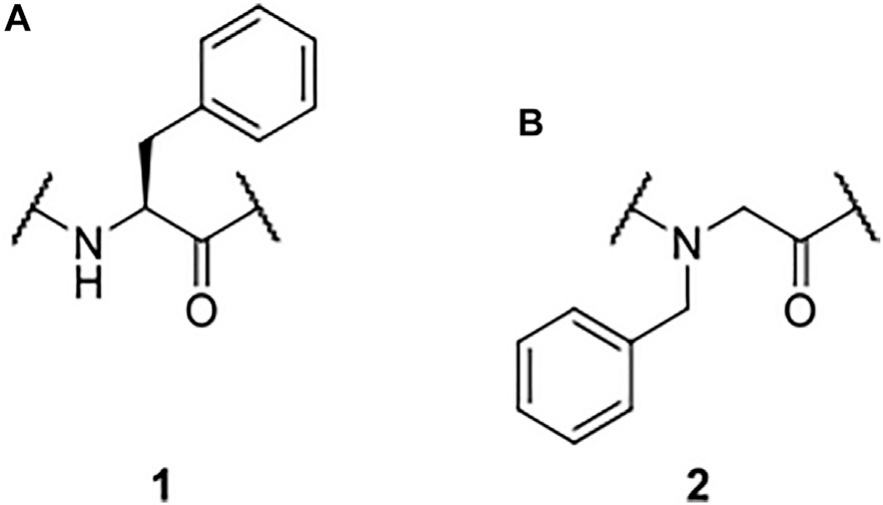
Phenylalanine (Green and Loewenstein, 1988) as an example for an amino acid as the building block of a peptide **(A)** and *N*1ph (Frankel and Pabo, 1988) as an example for an alkylated glycine monomer **(B)**.

In the late 1980s, the capability of the Trans-Activator of Transcription (Tat) protein of the Human Immunodeficiency Virus (HIV) to overcome cellular barriers without any receptor interaction was discovered. (Green and Loewenstein, 1988; Frankel and Pabo, 1988). Thereby, the foundation for a new compound class, the cell-penetrating peptides (CPPs), was laid. Since then, plenty of different CPPs capable to transport diverse cargos across cellular membranes have been published and outlined in numerous comprehensive reviews. (Koren and Torchilin, 2012; Copolovici et al., 2014; Guidotti et al., 2017; Kalafatovic and Giralt, 2017; Taylor and Zahid, 2020; Falanga et al., 2020; Desale et al., 2021) Although a lot of research is focused on CPPs, some questions remain open. As an example, the uptake mechanism of these molecular transporters is still not fully understood. (Richard et al., 2003; Madani et al., 2011; Ruseska and Zimmer, 2020). Thus, the efficiency enhancement of cellular uptake is one of many subjects of current research. (Nadal Bufí and Henriques, 2020; Fazil et al., 2020; Yin et al., 2020). It has already been shown that a rigidification of the spatial structure increases the activity of some CPPs due to improved interactions with the phospholipid bilayer, limited off-target effects, and a lower entropy penalty. (Lättig-Tünnemann et al., 2011; Nischan et al., 2015; Dougherty et al., 2019; Park et al., 2019) Moreover, the incorporation of certain peptidomimetics could enhance both cellular uptake efficiency and metabolic stability. (Kölmel et al., 2012; Huang et al., 2012; Jing et al., 2012; Vollrath et al., 2013; Koelmel et al., 2014; Dougherty et al., 2019) As CPPs resemble endogenous structures, they are prone to fast proteolytic degradation. (Goodwin et al., 2012; Zhang et al., 2020) Peptidomimetics such as peptoids mimic the structural and functional features of peptides while enhancing their bioavailability. (Dohm et al., 2011; Simon et al., 1992; Zuckermann et al., 1992; Zuckermann, 2011) Peptoids are *N*-substituted oligoglycines are readily accessible *via* well-established solid-phase synthesis techniques. (Zuckermann, 2011) Formally, they solely differ from peptides in the location of their side chain which is moved from the *α*-carbon to the nitrogen atom (Figure 1). However, this shift has a major impact on the conformational flexibility and, thus, on the spatial structure of peptoids (Yoo and Kirshenbaum, 2008; Zuckermann, 2011; Kirshenbaum and Zuckermann, 2019).

Besides the capability to form hydrogen bonds, the chirality at the *α*-carbon is lost when transforming peptides to peptoids. Interestingly, it has been shown that the absence of these features can even increase the efficiency of cellular uptake. (Wender et al., 2000; Goun et al., 2006; Tan et al., 2008; Schwochert et al., 2015) Together with their high stability against enzymatic degradation, cell-penetrating peptoids (CPPos) constitute promising tools for cargo delivery.

So far, mainly positively charged CPPos have been synthesized with cellular uptake specificity for endosomes, the cytosol, or the nucleus. (Murphy et al., 1998; Wender et al., 2000; Wright et al., 2003; Schröder et al., 2007; Schröder et al., 2008; Eggenberger et al., 2009; Unciti-Broceta et al., 2009; Huang et al., 2012; Kölmel et al., 2012; Sternberg et al., 2013; Kölmel et al., 2014; Marouseau et al., 2017) The incorporation of a critical amount of lipophilic side chains led to a specific accumulation in mitochondria. (Kölmel et al., 2012; Nam et al., 2018) It was stated, that a cationic charge may be a prerequisite for interaction with the negatively charged head groups of the phospholipid bilayer and subsequent cellular uptake through the plasma membrane. (Wender et al., 2000; Peretto et al., 2003; Madani et al., 2011; Koren and Torchilin, 2012; Guidotti et al., 2017) Shin *et al.* (Shin et al., 2018) showed that the efficiency of this cellular uptake is significantly increased after cyclization of linear CPPos.

As a defined three-dimensional structure caused by cyclization has proved to be beneficial for cellular uptake, (Lättig-Tünnemann et al., 2011; Nischan et al., 2015; Shin et al., 2018; Dougherty et al., 2019; Park et al., 2019), we herein report the synthesis of macrocyclic tetramers and hexamers as novel molecular transporters. The macrocycles are composed of both alkylated glycine monomers and amino acids to maintain steric information that may be crucial for high biological activity. (Agrawal et al., 2007; Henriques et al., 2019) These cyclic hybrids constitute a promising substance class combining the unique selectivity of peptides with the outstanding bioavailability of peptoids (Olsen, 2010; Schwochert et al., 2015).

## Results and Discussion

**SCHEME 1:**
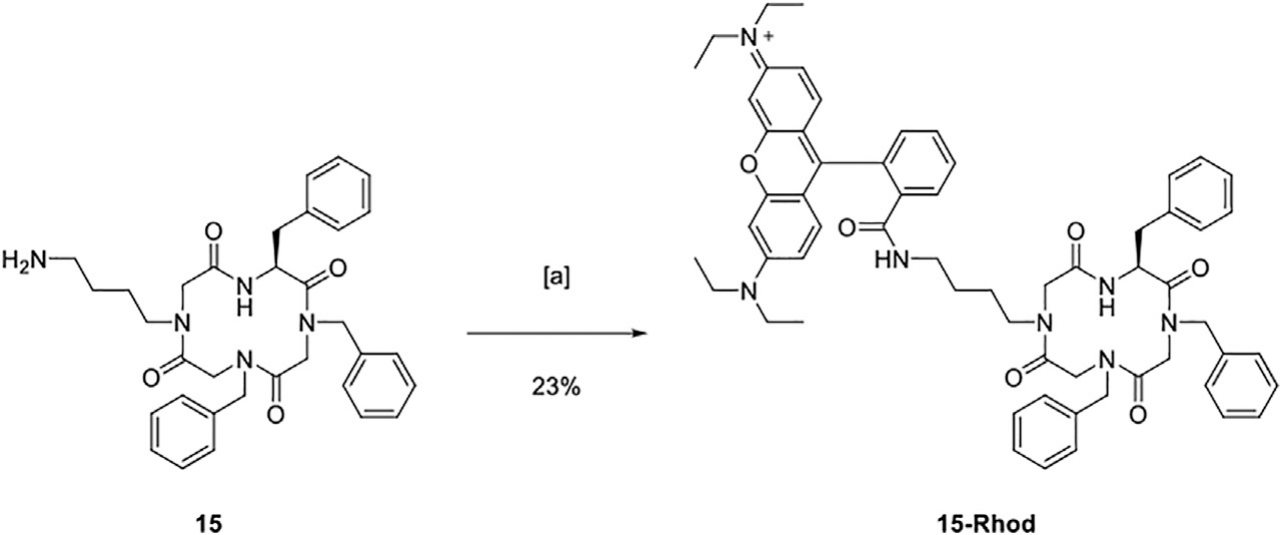
Synthetic approach for the assembly of linear precursors on solid support. **[A]**: Fmoc-protected amino acid, N,N′-diisopropylethylamine (DIPEA), N-methyl-2-pyrrolidone (NMP), 21°C, 16 h; **[B]**: piperidine, dimethylformamide (DMF), 21°C, 3 × 5 min; **[C]**: Fmoc-protected amino acid, N,N’-diisopropylcarbodiimide (DIC), hydroxybenzotriazole, NMP, 21°C, 4 h; **[D]**: bromoacetic acid, DIPEA, DMF, 21°C, 1 h; **[E]**: amine, DMF, 21°C, 1–16 h; **[F]**: bromoacetic acid, DIC, DMF, 21°C, 30 min; **[G]**: hexafluoroisopropanol, methylene chloride, 21°C, 16 h.

Macrocyclic tetramers and hexamers were synthesized in a multistep procedure using solid and solution phase methods. The synthetic approach involved the assembly of linear precursors on solid supports following the submonomer method published by Zuckermann (Zuckermann et al., 1992) as well as the solid phase peptide synthesis (SPPS) described by Merrifield (Merrifield, 1963). A 2-chlorotrityl chloride polystyrene resin (**3**) served as solid support (Scheme 1).

**FIGURE 2:**
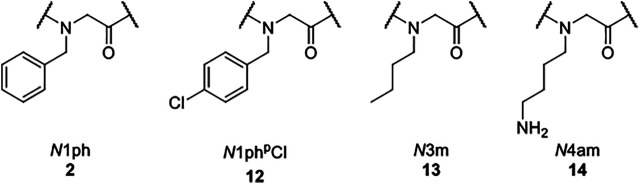
Alkylated glycine monomers 2 and 12–14 used for the synthesis of molecular transporters and their applied designation.

The submonomer method requires an acetylation step using a haloacetic acid followed by a substitution implementing the desired side chain. (Zuckermann et al., 1992) Thus, bromoacetic acid was either attached to the resin under basic conditions (→ **7**) or to the growing oligomer chain using diisopropylcarbodiimide (DIC) as a coupling reagent. A certain amine was added allowing for the assembly of an alkylated glycine monomer (→ **8**). The possibility to incorporate any amine gives rise to various side chains and, thus, easily tunable properties of the resulting peptoids. For the design of molecular transporters, the substitution step was performed with four different amines that led to the assembly of the alkylated glycine monomers *N*1ph (**2**), *N*1ph^p^Cl (**12**), *N*3m (**13**), and *N*4am (**14**, Figure 2). The former ones were empirically chosen to examine the influence of lipophilicity on the biological activity of the respective macrocycles. Thereby, all lipophilic monomers should allow for an accumulation in mitochondria. (Kölmel et al., 2012; Nam et al., 2018) The peptoid monomer *N*4am (**14**) served as conjugation site for the fluorophore Rhodamine B.

The SPPS was performed using Fmoc-protected amino acids. They were analogously attached to the resin (→ **4**) under basic conditions or to the growing linear precursor using DIC. To avoid racemization at the *α*-carbon, hydroxybenzotriazole was added for amino acid couplings. The temporary Fmoc-protecting group was cleaved with piperidine after coupling (→ **5**). The combination of both solid-phase methods enabled the straightforward synthesis of linear hybrids composed of alkylated glycine monomers as well as amino acids. The linear precursors **11** were cleaved from the resin under mildly acidic conditions releasing a carboxylic acid at the *C*-terminus.

**FIGURE 3:**
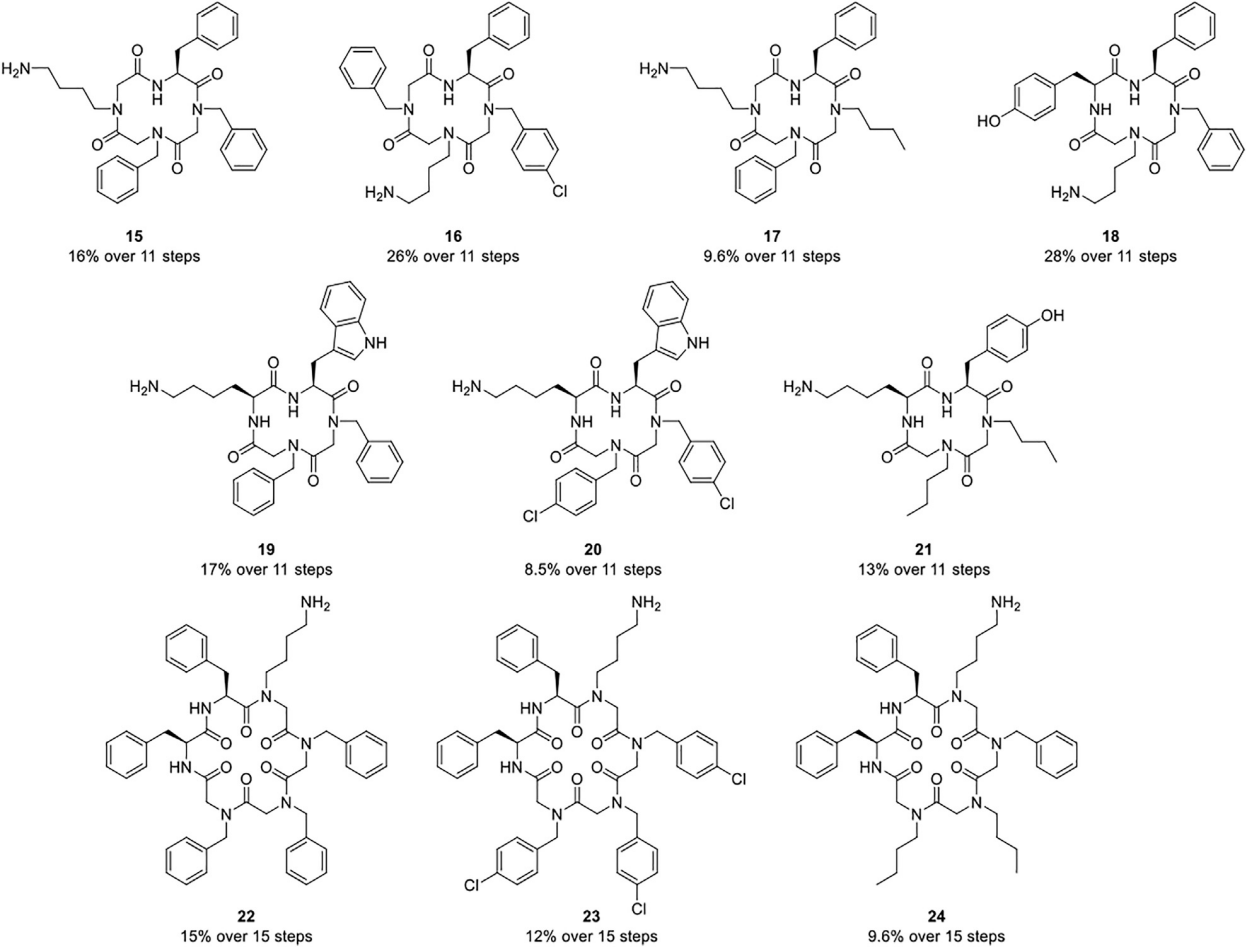
Macrocyclic hybrids 15–24 composed of alkylated glycine monomers and amino acids that were assembled *via* a combination of solid and solution phase methods.

The subsequent head-to-tail cyclization using the crude linear precursors and [dimethylamino(triazolo[4,5-b]pyridine-3-yloxy)methylidene]-dimethylazanium hexafluorophosphate (HATU) as potent coupling agent was performed under high dilution conditions to avoid favored side reactions like dimerizations. (Aldrich et al., 2011; Thakkar et al., 2013) Thus, the respective linear precursor was added dropwise to an extensively stirred solution of HATU. Thereby, the concentration of the added linear peptide-peptoid hybrid solution did not exceed 7.50 mm. After eleven or respectively fifteen reaction steps, the macrocycles were purified *via* preparative reversed-phase high performance liquid chromatography (HPLC). Product formation was confirmed *via* matrix-assisted laser desorption/ionization-time of flight (MALDI-TOF) mass spectrometry and subsequent nuclear magnetic resonance (NMR) spectroscopy. The synthetic protocol yielded the macrocyclic compounds **15**–**24** (Figure 3).

Seven cyclic tetramers (**15–21**) and three cyclic hexamers (**22–24**) were successfully synthesized. Every macrocycle is composed of several alkylated glycine monomers and up to two amino acids. The hybrids share aromatic as well as aliphatic residues and an aminobutyl side chain. In every case, an aromatic amino acid represents the *N-*terminus of the linear precursor. It was assumed that the higher nucleophilicity of a primary amine might be beneficial for the subsequent ring closure reaction.

Regardless of their size and composition, all macrocycles were isolated in similar yields (14% ± 5) and satisfying purities (94% ± 5). For the assembly of hybrids **15–17**, an alkylated glycine monomer was attached to the solid support. Compared to tetramers **18–21** that were immobilized *via* an amino acid, the yields of the respective linear precursors were slightly decreased: While the crude linear tetramers of macrocycles **18–21** were isolated in 70% ± 14 yields, the precursors of tetramers **15–17** yielded 41% ± 5. It is noteworthy, that either the attachment of an alkylated glycine monomer to the solid support or the number of alkylated glycine monomers might be decisive for this discrepancy. Cyclization occurred to be more efficient for hybrids **15–17**, possibly due to increased conformational flexibility, (Yoo et al., 2010; De Riccardis, 2020), resulting in similar overall yields.

As the attachment of an amino acid to the solid support proved to be more efficient, linear hexamers were immobilized *via* l-phenylalanine (**1**). Due to the additional reaction steps, the yields of the crude linear hexamers were lowered (49% ± 13). Nevertheless, every linear precursor was synthesized in a sufficient quantity for cyclization. Depending on the amount of the linear precursor isolated, cyclization was carried out by the addition of a 3.25–7.50 mm solution of the respective linear hybrid. However, no effects on the efficiency of the ring closure were observed within this concentration range.

**SCHEME 2:**
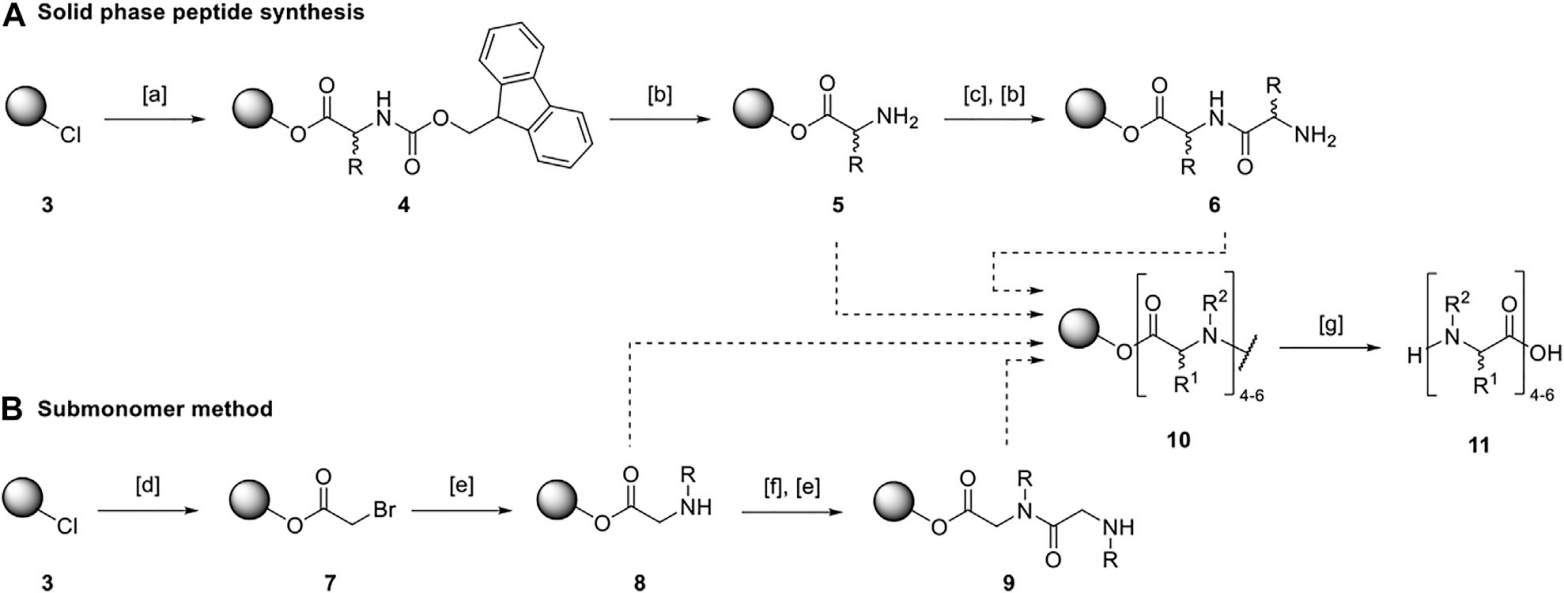
Conjugation of the chromophore Rhodamine B to a cyclic hybrid exemplarily shown for macrocycle 15. **[A]** Rhodamine B, HATU, DIPEA, DMF, 36 h, 21°C.

With the macrocyclic hybrids in hands, ten hydrophobic molecular transporters were synthesized. As a cargo, the potent fluorophore Rhodamine B was chosen. The chromophore is known for its biocompatibility and is widely used in biological research. (Lavis and Raines, 2008) Rhodamine B was attached *via* its carboxylic moiety to the aminobutyl side chains of the single macrocycles (Scheme 2).

The conjugation of Rhodamine B was conducted by the cyclization of linear precursors using HATU as a reliable activating agent. The labeling occurred in moderate yields (27% ± 5) and excellent purities (Table 1).

**TABLE 1:** Isolated yields and purities of the luminescent conjugates 15-Rhod to 24-Rhod.

Conjugate	Yield [%]	Purity [%]
15-Rhod	23	>99
16-Rhod	22	89
17-Rhod	17	>99
18-Rhod	7.0	93
19-Rhod	38	>99
20-Rhod	21	91
21-Rhod	11	>99
22-Rhod	52	>99
23-Rhod	62	>99
24-Rhod	14	>99

The cytotoxicity of the macrocyclic hybrids **15–24**, as well as their conjugates **15-Rhod** to **24-Rhod**, was examined using a standard MTT assay in human epithelial cervix carcinoma (HeLa) cells. This colorimetric assay displays the cell viability based on their capability to reduce the tetrazolium dye 3-(4,5-dimethylthiazol-2-yl)-2,5-diphenyltetrazolium bromide (MTT). (Mosmann, 1983; Tolosa et al., 2015) After treatment of HeLa cells with different concentrations of the respective macrocycles **15–24** (0.50─50 µm) for 72 h, no significant cytotoxic effects were observed. Most hybrids resulted in IC_50_ > 50 µm. However, in most cases the attachment of the chromophore Rhodamine B slightly increased the cytotoxicity of the respective molecular transporters **15-Rhod**–**24-Rhod** (Table 2). As shown by many research groups that Rhodamine B by itself is not efficiently entering the cells showing therefore an LD_50_ > 50 μM, we assume that this the toxicity of the Rhodamine moiety might be higher when increasingly localizing in the mitochondria to a higher amount.

**TABLE 2:** IC_50_ values of the macrocycles 15–24 as well as their conjugates 15-Rhod to 24-Rhod.

Macrocycle	IC_50_ [µm]	Conjugate	IC_50_ [µm]
15	>50	15-Rhod	>50
16	>50	16-Rhod	30
17	>50	17-Rhod	>50
18	>50	18-Rhod	33
19	>50	19-Rhod	34
20	>50	20-Rhod	>50
21	>50	21-Rhod	36
22	>50	22-Rhod	44
23	20	23-Rhod	6
24	>50	24-Rhod	26

Conjugate **23-Rhod** is the only hybrid with an IC_50_ value in a single-digit micromolar range (IC_50_ = 6 µm). Its parent macrocycle **23** turned out to be the most cytotoxic hybrid tested (IC_50_ = 20 µm). Considering every cyclic hybrid, the incorporation of the alkylated glycine monomer *N*1ph^p^Cl (**12**) was decisive for the cytotoxicity of the respective macrocycles: the higher the amount of *N*1ph^p^Cl building blocks, the lower the viability of the HeLa cells after treatment. However, comparing the macrocyclic transporters **19-Rhod** and **20-Rhod**, the substitution of two *N*1ph (**2**) monomers by two *N*1ph^p^Cl building blocks led to a decrease of cytotoxicity.

As **15-Rhod** to **24-Rhod** were not toxic at 5 µM the following transport experiments were carried out using this concentration. To study their cellular uptake and organellar localization, HeLa cells were treated with 5 µM of the cyclic conjugates **15-Rhod** to **24-Rhod** for 5 h and eventually imaged using confocal microscopy (for experimental details, see supplemental Material).

**FIGURE 4:**
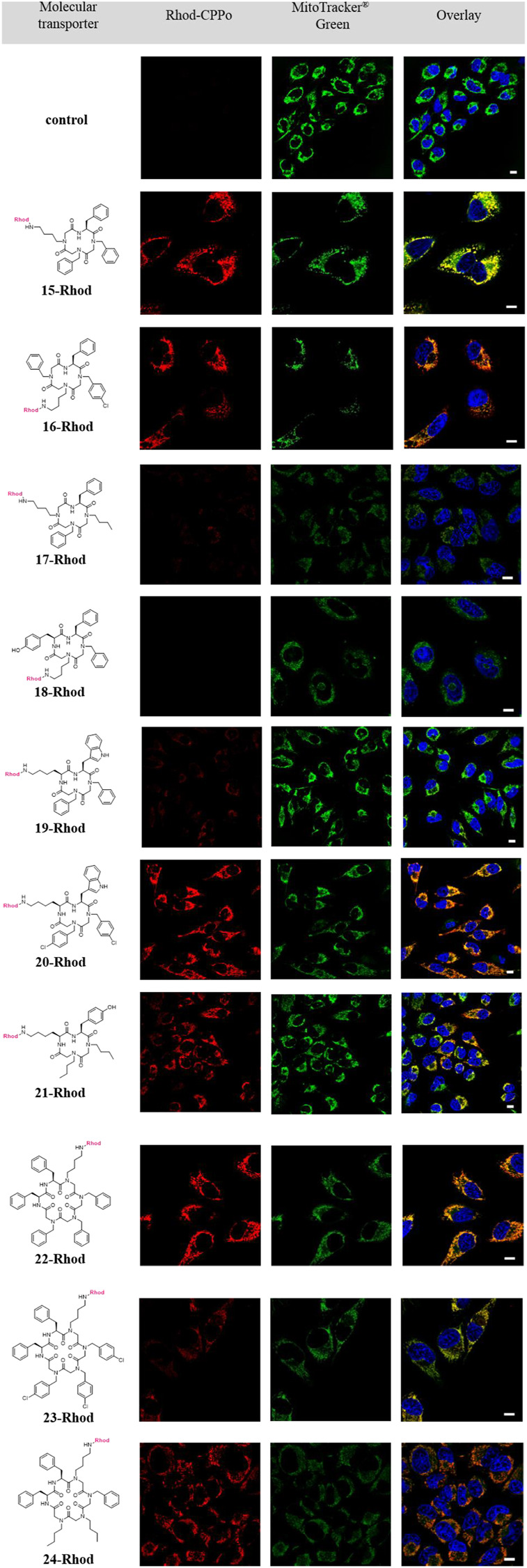
Localization of the macrocyclic conjugates 15-Rhod to 24-Rhod in HeLa cells. After incubation with a 5.0 µm solution of the respective transporters for 5 h, the cellular uptake was monitored *via* fluorescent confocal microscopy (Rhodamine B labeled hybrids: *λ*_Exc_ = 532 nm, *λ*_Em_ = 570–620 nm; MitoTracker^®^ Green: *λ*_Exc_ = 488 nm, *λ*_Em_ = 490–540 nm; Hoechst 33,342: *λ*_Exc_ = 405 nm, *λ*_Em_ = 430–490 nm). Counterstaining with MitoTracker^®^ Green visualized mitochondria (green). Staining with 2 μg/ml Hoechst 33,342 indicated nuclei (blue). Scale bar: 10 µm.

The molecular transporters **15-Rhod, 16-Rhod**, **20-Rhod-22-Rhod,** and **24-Rhod** were efficiently taken up by the cells and localized to different organelles due to their differences in their side-chain corona. Counterstaining of mitochondria using the mitochondrial marker MitoTracker Green^®^ gave evidence of a mitochondrial accumulation for conjugates **15-Rhod** to **17-Rhod** and **19-Rhod** to **24-Rhod** (Figure 4).

A high cellular uptake efficiency, as well as mitochondrial specificity, was observed for the molecular transporters **15-Rhod, 16-Rhod**, **20-Rhod** to **22-Rhod,** and **24-Rhod**. Mitochondrial accumulation was also shown for conjugates **17-Rhod**, **19-Rhod** and **23-Rhod**, however, their cellular uptake was significantly less efficient. An interesting discrepancy in the uptake efficiency was observed when comparing the molecular transporters **18-Rhod** and **21-Rhod** which are both characterized by the amino acid l-tyrosine. While **18-Rhod** containing two benzyl side chains was not internalized by the HeLa cells, conjugate **21-Rhod**, in which both benzyl moieties were replaced by two aliphatic side chains, was efficiently taken up. By comparison of the cyclic tetramers **15-Rhod** and **17-Rhod** as well as the hexamers **22-Rhod** and **24-Rhod**, the substitution of aromatic residues by aliphatic side chains had no positive effect on the cellular efficiency. **17-Rhod** was even less internalized than **15-Rhod**. Regarding substitutions of *N*1ph (**2**) by its halogenated relative *N*1ph^p^Cl (**13**), no conclusive effects were observed as well. Conjugates **15-Rhod** and **16-Rhod** showed a similar cellular uptake efficiency, while an exchange of two *N*1ph (**2**) side chains by *N*1ph^p^Cl (**13**) increased the uptake of the molecular transporter **20-Rhod** compared to conjugate **19-Rhod**. However, the halogenation of three *N*1ph (**2**) building blocks led to a significantly decreased cellular uptake efficiency which becomes evident by comparison of hexamers **22-Rhod** and **23-Rhod**.

The molecular transporters **15-Rhod**, **20-Rhod** and **22-Rhod** are characterized by low cytotoxicity, efficient cellular uptake, and high specificity. Thus, they represent the most promising cyclic hybrids capable of the transport of different cargos into mitochondria. The data obtained support previous studies on peptides and peptoids indicating that a certain amount of lipophilic residues may be crucial for mitochondrial targeting. (Kölmel et al., 2012; Nam et al., 2018) Besides, the data prove that no cationic charges are needed for mitochondrial localization. The capability of hydrophobic structures to function as molecular transporters is also known for so-called hydrophobic CPPs. However, only a few members of this substance class have been discovered yet. (Xie et al., 2020; Desale et al., 2021) It is assumed, that hydrophobic CPPs may penetrate cellular membranes in a direct, energy-independent manner dispensing with endocytosis. (Gao et al., 2002; Gao et al., 2011; Nakayama et al., 2011; Guidotti et al., 2017; Tian et al., 2017) The latter leads to a major challenge as molecular transporters might not escape the endosomes and get degraded instead of reaching their designated targets. (Nadal Bufí and Henriques, 2020) As exemplaryly shown for the macrocyclic transporter **22-Rhod**, the mitochondrial uptake is occurring at 37°C as well as at 4°C (see supplemental material, Supplementary Figure S1). The efficient internalization at a low temperature led us to the assumption that **22-Rhod** might directly penetrate the cell membrane. However, the uptake mechanism of macrocyclic hybrids will be further examined.

The versatile substance class of peptide-peptoid macrocycles shows high potential as molecular transporters. Their specific targeting of mitochondria paves the way for the treatment of various diseases that are associated with these organelles. (Malty et al., 2015; Javadov et al., 2020; Tan and Finkel, 2020) Structural studies on macrocyclic hybrids as well as an establishment of a comprehensive structure-activity relationship will henceforth enlarge their applicability as versatile tools in pharmaceutical and basic research.

## Conclusion

Ten macrocyclic peptide-peptoid hybrids were synthesized in a straightforward approach using solid and liquid phase techniques. Their unique structure combines the advantages of both peptides and peptoids. The conformational constraints introduced by chiral amino acids as well as the cyclization of their backbone render them promising model structures for selective compounds. Their capability to form hydrogen bonds makes them soluble in an aqueous environment. Furthermore, the incorporation of *N*-alkylated glycine monomers resulting in a hybrid structure increases their polarity and variability by allowing for the incorporation of plenty of different side chains and easily tunable properties. The hydrophobic molecular transporters presented selectively accumulated in mitochondria without the need for additional cationic charges. Thus, their uptake mechanism might differ from known CPPs and CPPos. Further studies on these versatile structures will reveal their potential as selective and highly efficient molecular transporters.

## Data Availability

The data presented in this study are openly available in the Chemotion repository: www.chemotion-repository.net.
