# Supplementary material for: Cyclic Peptoid-Peptide Hybrids as Versatile Molecular Transporters
Source: Front Chem. 2021 Jun 25;9:696957. doi: 10.3389/fchem.2021.696957 (PMC8267177; doi:10.3389/fchem.2021.696957)
Supplement: Supplementary file 1 [file DataSheet1.DOCX]

SUPPORTING INFORMATION

General experimental details

**High-performance liquid chromatography (HPLC)**

Reversed-phase analytical HPLC was performed on an Agilent Series 1100 equipped with a G1322A degasser, a G1311A pump, a G1313A autosampler, a G1316A oven, and a G1315B diode array detector (DAD). Alternatively, the HPLC system Thermofisher Ultimate 3000_1, equipped with an LPG-3400SD pump, a WPS-3000 autosampler, a TCC-3000 oven, and a DAD-3000 diode array detector was used. As a stationary phase, a VDSpher® C18-M-SE column (5.00 µm, 250 × 4.00 mm, VDS Optilab) was used. As the mobile phase, a linear gradient of 5–95% acetonitrile in double-distilled water with 0.1% trifluoroacetic acid (TFA) with a flow rate of 1 mL/min over 30 min (unless stated otherwise) was used. Purity was calculated by integration of the detected signals at 218 nm.

Reversed-phase preparative HPLC was performed using the Interchim HPLC system Puriflash™ 4125, equipped with the software InterSoft® V5.1.08. As a stationary phase, a VDSpher® C_18_-M-SE precolumn (10 µm, 40 x 16 mm, VDS Optilab), followed by a VDSpher® C_18_-M-SE column (10 µm, 250 x 20 mm, VDS Optilab) was used. As a mobile phase, a gradient of acetonitrile in double-distilled water with 0.1% TFA with a flow rate of 15 mL/min was used. The method (gradient, duration) was adjusted to each sample. Detection was carried out with a UV diode array detector in a spectral range of λ = 200–600 nm. Fractions absorbing at 218 nm, 256 nm, or 280 nm were isolated.

**Matrix-Assisted Laser Desorption Ionization Time of Flight Mass Spectrometry (MALDI-TOF MS)**

MALDI-TOF mass spectra were measured on a Shimadzu Biotech Axima Confidence (model TO-6071R00) spectrometer, equipped with the software Shimadzu Biotech Launchpad™ (version 2.9.3.20110624). For desorption, a nitrogen laser (λ = 337 nm) was used. The samples were spotted on a Shimadzu Kratos analytical standard stainless steel target (DE1580TA) with 386 spots. As a matrix, a commercially available 1:1 mixture of 2,5-dihydroxybenzoic acid (DHB) and α-cyano-4-hydroxycinnamic acid (CHCA) (Universal MALDI matrix from Sigma-Aldrich®) was used as a saturated solution in 50% acetonitrile in double-distilled water. For every spectrum, the samples were shot around 100 times with a frequency of 50 Hz. The protonated molecule ion is expressed as [M+H]^+^, pseudo-molecule ions as [M+Na]^+^, and [M+K]^+^, respectively.

**Nuclear Magnetic Resonance Spectroscopy (NMR)**

NMR spectra were recorded at 25 °C on a Bruker Avance 300 (300 MHz (^1^H), 75 MHz (^13^C)) and a Bruker Avance DRX 500 (500 MHz (^1^H), 125 MHz (^13^C)) spectrometer. Deuterated solvents were purchased from Eurisotop. Chemical shifts (δ) are expressed in parts per million (ppm). All spectra were referenced to the signals of the residual protons of the solvents acetonitrile‑*d*_3_ (1.94 ppm (^1^H), 118.3 ppm (^13^C)), dimethylsulfoxide-*d*_6_ (2.50 ppm (^1^H), 39.5 ppm (^13^C)) or methanol-*d*_4_ (3.31 ppm (^1^H), 49.0 ppm (^13^C)) as internal standards. The spectra were analyzed according to the first order. Coupling constants (*J*) are given in Hertz (Hz). Multiplicities of signals are described as follows: s = singlet, d = doublet, t = triplet, q = quartet, quin = quintet, m = multiplet. Abbreviations for signal assignments include H_Ar_ = aromatic proton, C_Ar_ = aromatic carbon. ^1^H and ^13^C signals were assigned with the help of ^1^H-^13^C-HSQC (Heteronuclear Single Quantum Coherence) and ^1^H-^1^H-COSY (Correlation Spectroscopy) experiments. Phase-resolved ^1^H-^13^C-HSQC was used for the assignment of ^13^C signals. Primary as well as tertiary carbon atoms are indicated by a “+”, secondary ones are marked with a “–“, whilst quaternary carbons are indicated by “C_q_”.

Biological assays

Biological assays were carried out under sterile conditions using the sterile benches LaminAir® HB2448 (Heraeus GmbH), Laminar-Flow Werkbank (BDK Luft- und Reinraumtechnik GmbH) or Clean Air Technik (Clean Air). Cells were incubated at 37 °C, 5% CO_2_, and 85% humidity using a Binder Ex-Demo Units Incubator.

**Cell culture**

Human epithelial cervix carcinoma (HeLa) cells were cultivated in *Dulbecco ́s Modified Eagle Medium-high glucose* (DMEM, Gibco^TM^) supplemented with 10% fetal calf serum (FCS) and 1% penicillin/streptomycin (P/S). At 80% confluency, the medium was removed, the cells were washed with *Dulbecco’s Phosphate Buffered Saline* (DPBS, Gibco^TM^), treated with trypsin / EDTA (Gibco^TM^) to passage the cells. The detachment process is stopped by DMEM containing FCS and the cells were seeded according to the desired cell number/area.

**MTT-assay^(1)^**

In a 96-well plate (Corning® Costar® 3596 96-Well Cell Culture Cluster), 1.0 × 10^4^ HeLa-cells per well of a 96 well plate were cultivated for 16 h. Stock solutions of the single compounds in dimethylsulfoxide were diluted to 0.50 µm, 5.00 µm, and 50.0 µm with cell culture medium (DMEM + 10% FBS + 1% P/S) to ensure a final concentration of DMSO below 0.5%. 100 µL of the prepared DMEM diluted compounds were added to each well and the cells were incubated for 72 h. All experiments were performed in triplicates. Cells that were solely treated with the culture medium containing 0.5% of DMSO served as a negative control. After incubation, the positive control (cells in DMSO plus 0.5% DMSO) was treated with 1% Triton-X. To determine the viability of the cells 20 µL of a freshly prepared solution of 3-(4,5-dimethylthiazol-2-yl)-2,5-diphenyltetrazolium bromide (MTT, 2.73 mg/mL, Sigma-Aldrich®) in culture medium were added to each well and incubated at 37 °C for 4 h. The reaction was stopped using 100 µL of *Solubilization Solution / Stop Mix* (CellTiter 96® Non-Radioactive Cell Proliferation Assay, Promega). After 24 h, the absorption (A) of every well at 595 nm was monitored using a 96-well plate reader (Ultra Microplate Reader ELx808, Biotech Instruments Inc.).

**Localization assay**

In an 8-well chamber slide (µ-Slide 8-Well ibidiTreat, ibidi®), 1.0 × 10^4^ HeLa cells per well were cultivated for 16 h. The cyclic hybrids were diluted with DMEM to yield a 5 µM concentration. The cells were treated with the hybrids for 5 h at 37 °C. To monitor the intracellular localization, the cells were counterstained with MitoTracker® (Invitrogen) and 2 µg/mL Hoechst 33342. After washing with DPBS (3 ×), the luminescence was monitored *via* life-cell imaging using a Leica TCS-SPE confocal microscope (DM2500 inverse microscope, 8 Bit resolution, 1024 × 1024 pixels), equipped with a 1 Airy pinhole aperture (111.5 µm), ACS APO 63.0x/1.30 OIL UV and 40x/1.14 OIL UV objectives and the images were processed by the Leica Application Suite (LAS-AF) Version 2.1.2 build 4530 software. Images were taken with a scanning frequency of 400 Hz. Background noise was averaged by an eight-fold repetition of the scan process. The cyclic hybrids were excited at 532 nm, MitoTracker® Green at 488 nm, and Hoechst 33342 at 405 nm. The exposure was set to minimize oversaturated pixels in the final images. Fluorescence emission was measured at 570–620 nm for the detection of rhodamine B labeled hybrids, at 490–540 nm for the detection of mitochondria, and 430–490 nm for the detection of nuclei.


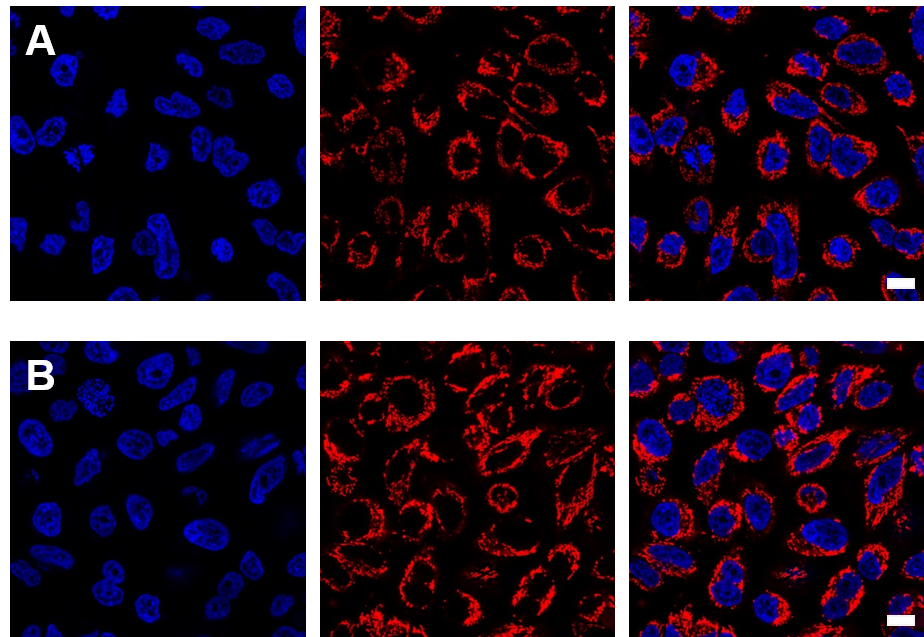


**Figure S1:** Confocal Laser Scanning Microscopy **(**CLSM) images of HeLa cells after 2 h incubation with 5 µM peptoid **22-Rhod** at 4 °C (top line, A) and 37 °C (bottom line, B). Blue emission of cell nuclei stained with Hoechst 33342 (λ_Exc_ = 405 nm, λ_Em_ = 420-547 nm), red emission of rhodamine B labelled peptoid **15-Rhod** (λ_Exc_ = 542 nm, λ_Em_ = 547-750 nm), merge. Scale 10 µm.

General experimental procedures

Solvents and reagents were purchased from commercial sources and used without further purification. Water was deionized using an Elix® Essential 15 water purification system (Millipore®) and purified further with a Milli-Q Biocel system (Q-Gard® 1, Quantum® X, Millipore®) for chromatographical applications. Abbreviations are as follows: acetonitrile (ACN), *^tert^*butyloxycarbonyl (Boc), *N,N’*‑diisopropylcarbodiimide (DIC), *N,N’*-diisopropylethylamine (DIPEA), *N,N’*-dimethylformamide (DMF), fluorenylmethoxycarbonyl (Fmoc), [dimethylamino(triazolo[4,5-b]pyridine-3-yloxy)methylidene]-dimethylazanium hexafluorophosphate (HATU), 1,1,1,3,3,3‑hexafluoroisopropyl alcohol (HFIP), 1-hydroxybenzotriazole (HOBt), methylene chloride (DCM), *N*-methylpyrrolidone (NMP), trifluoroacetic acid (TFA).

Reagents and products were weighed on a Sartorius CP224S (d = 0.1 mg) or a Radwag AS 220.X2 (d = 0.1 mg) scale.

Solvents were removed under reduced pressure on a rotary evaporator at 40–60 °C heating bath temperature. Aqueous solutions were deep-frozen and lyophilized under reduced pressure on an LDC-1 (Alpha 2–4) dry freezer (Christ).

Solid-phase synthesis(2, 3)

Solid-phase synthesis of linear precursors was performed by the published solid-phase peptide synthesis(2) and the submonomer technique(3) in 6 mL plastic-fritted syringes (Multisynthec GmbH), closed with a plastic cap. As solid support, a 2‑chlorotrityl chloride resin (Carbolution, 1.60 mmol/g loading density, 100–200 mesh,) was used. Reaction steps were performed on a KS501 digital circular shaker (160–200 rpm, IKA-Labortechnik) at 21 °C. Yields were calculated according to the resin loading value.

General Procedure (GP1) for the immobilization of an amino acid(2)

In a fritted syringe, the 2-chlorotrityl chloride resin (125 mg, 200 µmol, 1.60 mmol/mg loading density, 1.00 equiv.) was swollen in 2.50 mL of DCM for at least 30 min. After filtration, the respective Fmoc-protected amino acid (800 µmol, 4.00 equiv.) and DIPEA (139 µL, 103 mg, 800 µmol, 4.00 equiv.) were dissolved in 2.00 mL of NMP and added to the resin. The reaction mixture was incubated overnight at 21 °C. The solvent was removed by filtration and the resin was washed with 2.00 mL of DMF, methanol, DCM, and DMF each.

Afterward, the resin was incubated with 2.50 mL of a 20% solution of piperidine in DMF for 5 min. The solvent was removed and the procedure was repeated twice. The resin was washed as described.

General Procedure (GP2) for the immobilization of a peptoid monomer(3)

In a fritted syringe, the 2-chlorotrityl chloride resin (125 mg, 200 µmol, 1.60 mmol/mg loading density, 1.00 equiv.) was swollen in 2.50 mL of DCM for at least 30 min. After filtration, a 1 m solution of bromoacetic acid (222 mg, 1.60 mmol, 8.00 equiv.) and DIPEA (278 µL, 206 mg, 1.60 mmol, 8.00 equiv.) in DMF was added to the resin. The reaction mixture was incubated for 1 h at 21 °C. The solvent was removed by filtration and the resin was washed with 2 mL of DMF, methanol, DCM, and DMF each.

Subsequently, a solution of the respective amine (1.60 mmol, 8.00 equiv.) in 2 mL of DMF was added and the mixture was shaken for 1 h at 21 °C. The solvent was removed and the resin was washed as described.

General Procedure (GP3) for the coupling of an amino acid(2)

The respective Fmoc-protected amino acid (800 µmol, 4.00 equiv.) and HOBt (123 mg, 800 µmol, 4.00 equiv.) were dissolved in 2 mL of NMP. DIC (125 µL, 101 mg, 800 µmol, 4.00 equiv.) was added and the resin was immediately incubated with the resulting solution for 4 h at 21 °C. The solvent was removed by filtration and the resin was washed as described.

Afterward, the resin was incubated with 2.50 mL of a 20% solution of piperidine in DMF for 5 min. The solvent was removed and the procedure was repeated twice. The resin was washed as described.

General Procedure (GP4) for the coupling of a peptoid monomer(3)

DIC (250 µL, 202 mg, 1.60 mmol, 8.00 equiv.) was added to a 1 m solution of bromoacetic acid (222 mg, 1.60 mmol, 8.00 equiv.) in DMF. The solution was immediately added to the resin and shaken for 30 min at 21 °C. The solvent was removed by filtration and the resin was washed as described.

Subsequently, a solution of the respective amine (1.60 mmol, 8.00 equiv.) in 2 mL of DMF was added and the mixture was shaken for 1 h at 21 °C. The solvent was removed and the resin was washed as described.

General Procedure (GP5) for the cleavage of linear precursors

The resin was extensively washed with DCM. 2.50 mL of a solution of 33% HFIP in DCM was added and incubated twice for 1 h or overnight, respectively, at 21 °C. The cleavage cocktail was collected and the resin was flushed with DCM. The solvent was removed under the air stream. The residue was dissolved in acetonitrile and water, deep-frozen, and lyophilized to give the crude linear precursor.

General Procedure (GP6) for the cyclization of linear precursors(4)

HATU (0.750 equiv.) was dissolved in 30 mL of DMF. In parallel, the linear precursor (1.00 equiv.) was dissolved in 20 mL of DMF, and DIPEA (8.00 equiv.) was added. 10 mL of this mixture were added to the extensively stirred HATU solution over a period of 6 h at 21 °C. Afterward, an additional 0.750 equiv. of HATU were added to the reaction mixture in one portion. The remaining 10 mL of the linear precursor mixture were added over another period of 6 h. The reaction was stirred for 12 h at 21 °C and the solvent was removed under reduced pressure.

General Procedure (GP7) for the cleavage of Boc- and *^tert^*butyl-protecting groups

The residue was dissolved in 5 mL of a 95% solution of TFA in DCM and stirred for 1 h at 21 °C. The solvent was removed under the air stream.

General Procedure (GP8) for the attachment of rhodamine B

The respective hybrid (1.00 equiv.), HATU (1.50 equiv.), rhodamine B (1.50 equiv.), and DIPEA (8.00 equiv.) was dissolved in DMF and stirred for 36 h at 21 °C. The solvent was removed under reduced pressure.

*N‑*Boc-diaminobutane (25)(5)

1,4-diaminobutane (57.2 g, 649 mmol, 6.50 equiv.) was dissolved in 400 mL of DCM. Over a period of 6 h, a solution of di-*^tert^*butyl dicarbonate (21.8 g, 99.9 mmol, 1.00 equiv.) in 220 mL of DCM was added under extensive stirring at 21 °C. The reaction mixture was stirred overnight, the solvent was removed under reduced pressure and the residue was dissolved in 30 mL of cold water. The solution was filtered and extracted using ethyl acetate (4 ×). The combined organic layers were washed with water (2 ×) and brine, dried over Na_2_SO_4_ and the solvent was removed under reduced pressure. *N‑*Boc-diaminobutane (**25**) was isolated as a colorless oil (16.2 g, 86.1 mmol, 86%).

**^1^H-NMR** (300 MHz, chloroform-*d_1_*, ppm): δ = 4.75 (bs, 1H, N*H*), 3.06 (q, *J* = 6.3 Hz, 2H, C*H*_2_NHCO), 2.65 (t, *J* = 6.4 Hz, 2H, C*H*_2_NH_2_), 1.40–1.49 (m, 4H, 2 x C*H*_2_), 1.37 (s, 9H, 3 x C*H*_3_), 1.29 (s, 2H, N*H*_2_). Analytical data comply with literature. (5)

*cyclo*-(l‑Phe-*N*1ph-*N*1ph-*N*4am) (15)

Following **GP2**, the peptoid monomer *N*4am was immobilized using *N*-Boc-diaminobutane (**25**, 310 mg, 1.60 mmol, 8.00 equiv) for substitution. The subsequent peptoid monomers *N*1ph were incorporated following **GP4** using benzylamine (174 µL, 171 mg, 1.60 mmol, 8.00 equiv.) as a submonomer. Subsequently, Fmoc-l‑phenylalanine (310 mg, 800 µmol, 4.00 equiv.) was coupled following **GP3**. Cleavage was performed by **GP5**. The crude linear precursor (103 mg, 150 µmol, 1.00 equiv.) was cyclized following **GP6** and deprotected according to **GP7**. After purification *via* preparative reversed-phase HPLC (5–95% acetonitrile in water with 0.1% TFA in 45 min), the macrocycle was isolated as a colorless solid (30.8 mg, 54.1 µmol, 16% over 11 steps).

The NMR spectra contain multiple signal sets. Chemical shifts of the most prominent isomer are given. **^1^H-NMR** (500 MHz, acetonitrile‑*d_3_*, ppm): δ = 7.39–7.01 (m, 15H, 5 ×phe-C*H*, 10 × *N*1ph-C*H*), 6.82 (d, *J* = 10.4 Hz, 1H, phe-N_α_*H*), 5.39 (d, *J* = 15.0 Hz, 1H, COC*H*HN), 5.32 (d, *J* = 14.6 Hz, 1H, COC*H*HN), 5.02 (td, *J* = 9.4, 5.7 Hz, 1H, phe-C_α_*H*), 4.56 (d, *J* = 14.6 Hz, 1H, *N*1ph-C*H*H), 4.38 (d, *J* = 17.6 Hz, 1H, *N*1ph-C*H*H), 4.24 (d, *J* = 14.6 Hz, 1H, *N*1ph-CH*H*), 4.05 (d, *J* = 18.2 Hz, 1H, COC*H*HN), 3.94–3.87 (m, 2H, COCH*H*N, *N*1ph-CH*H*), 3.64 (d, *J* = 18.3 Hz, 1H, COCH*H*N), 3.45–3.35 (m, 1H, *N*4am-C_1_*H*H), 3.27–3.19 (m, 1H, phe-C*H*H), 3.11 (d, *J* = 14.5 Hz, 1H, COCH*H*N), 2.97–2.85 (m, 2H, *N*4am-C_4_*H*_2_), 2.82–2.71 (m, 4H, phe-CH*H*, *N*4am-C_1_*H*H, *N*4am-N*H*_2_), 1.60 (p, *J* = 7.3 Hz, 2H, *N*4am-C_3_*H*_2_), 1.54–1.42 (m, 2H, *N*4am-C_2_*H*_2_). – **^13^C-NMR** (125 MHz, acetonitrile‑*d_3_*, ppm): δ = 171.2 (C_q_, *C*ON), 170.7 (C_q_, *C*ON), 169.3 (C_q_, *C*ON), 168.3 (C_q_, *C*ON), 139.4 (C_q_, *C*_ar_), 138.0 (C_q_, *C*_ar_), 136.7 (C_q_, *C*_ar_), 131.1 (+, *C*_ar_H), 130.5 (+, *C*_ar_H), 129.7 (+, *C*_ar_H), 129.5 (+, *C*_ar_H), 129.4 (+, *C*_ar_H), 129.3 (+, *C*_ar_H), 129.2 (+, *C*_ar_H), 129.0 (+, *C*_ar_H), 128.9 (+, *C*_ar_H), 128.6 (+, *C*_ar_H), 128.5 (+, *C*_ar_H), 128.2 (+, *C*_ar_H), 127.9 (+, *C*_ar_H), 127.8 (+, *C*_ar_H), 127.1 (+, *C*_ar_H), 51.9 (+, phe-*C*_α_H), 51.6 (–, CO*C*H_2_N), 50.7 (–, CO*C*H_2_N), 50.0 (–, *N*1ph-*C*H_2_), 49.3(–, *N*1ph-*C*H_2_), 49.1 (–, *N*4am-*C*_1_H_2_), 48.1 (–, CO*C*H_2_N), 40.1 (–, *N*4am-*C*_4_H_2_), 38.4 (–, phe-*C*H_2_), 24.9 (–, *N*4am-*C*_2_H_2_), 24.7 (–, *N*4am-*C*_3_H_2_). – **Analytical HPLC** (5–95% acetonitrile + 0.1% TFA in 30 min, detection at 218 nm): t_Ret_ = 11.2 min (>99%). – **MS**(m/z, MALDI-TOF): 570 [M+H]^+^, 592 [M+Na]^+^, 608 [M+K]^+^.

*cyclo*-(l‑Phe-*N*1ph-*N*1ph-*N*4am(Rhod)) (15-Rhod)

Macrocycle **15** (14.8 mg, 26.0 µmol, 1.00 equiv.) was conjugated to rhodamine B following **GP8**. After purification *via* preparative reversed-phase HPLC (5–95% acetonitrile in water with 0.1% TFA in 35 min), the title compound was isolated as a pink solid (6.00 mg, 6.03 µmol, 23%).

**Analytical HPLC** (5–95% acetonitrile + 0.1% TFA in 30 min, detection at 218 nm): t_Ret_ = 13.1 min (>99%). – **MS**(m/z, MALDI-TOF): 995 [M+H]^+^, 1017 [M+Na]^+^.

*cyclo*-(l‑Phe-*N*1ph^p^Cl-*N*4am-*N*1ph) (16)

Following **GP2**, the peptoid monomer *N*1ph was immobilized using benzylamine (174 µL, 171 mg, 1.60 mmol, 8.00 equiv.) for substitution. The subsequent peptoid monomers *N*4am and *N*1ph^p^Cl were incorporated following **GP4** using *N*-Boc-diaminobutane (**25**, 310 mg, 1.60 mmol, 8.00 equiv) and *p*-chlorobenzylamine (195 µL, 227 mg, 1.60 mmol, 8.00 equiv.) as submonomer, respectively. Subsequently, Fmoc-l‑phenylalanine (310 mg, 800 µmol, 4.00 equiv.) was coupled following **GP3**. Cleavage was performed by **GP5**. The crude linear precursor (66.5 mg, 92.1 µmol, 1.00 equiv.) was cyclized following **GP6** and deprotected according to **GP7**. After purification *via* preparative reversed-phase HPLC (5–95% acetonitrile in water with 0.1% TFA in 45 min), the macrocycle was isolated as a colorless solid (31.7 mg, 52.5 µmol, 26% over 11 steps).

The NMR spectra contain multiple signal sets. Chemical shifts of the most prominent isomer are given. **^1^H-NMR** (500 MHz, acetonitrile‑*d_3_*, ppm): δ = 7.43–7.11 (m, 14H, 5 × phe-C*H*, 5 × *N*1ph-C*H*, 4 × *N*1ph^p^Cl-C*H*) 6.72 (d, *J* = 8.6 Hz, 1H, phe-N_α_*H*), 5.34 (d, *J* = 15.2 Hz, 1H, COC*H*HN), 5.25 (d, *J* = 14.7 Hz, 1H, COC*H*HN), 5.22 (d, *J* = 15.5 Hz, 1H, COC*H*HN), 4.99 (td, *J* = 9.6, 5.6 Hz, 1H, phe-C_α_*H*), 4.32 (d, *J* = 18.0 Hz, 1H, *N*1ph^p^Cl-C*H*H), 3.86 (d, *J* = 15.5 Hz, 1H, COCH*H*N), 3.83 (d, *J* = 18.1 Hz, 1H, *N*1ph^p^Cl-CH*H*), 3.69–3.61 (m, 2H, COCH*H*N, *N*1ph-C*H*H), 3.39 (d, *J* = 17.4 Hz, 1H, *N*1ph-CH*H*), 3.35 (d, *J* = 14.6 Hz, 1H, COCH*H*N), 3.21–3.09 (m, 2H, phe-C*H*H, *N*4am-C_1_*H*H), 2.92-2.86 (m, 4H, *N*4am-C_4_*H*_2_, *N*4am-N*H*_2_), 2.85–2.76 (m, 1H, *N*4am-C_1_H*H*), 2.76–2.69 (m, 1H, phe-CH*H*), 1.70–1.61 (m, 1H, *N*4am-C_2_*H*H), 1.59–1.43 (m, 3H, *N*4am-C_2_H*H*, *N*4am-C_3_*H*_2_). – **^13^C-NMR** (125 MHz, acetonitrile‑*d_3_*, ppm): δ = 171.6 (C_q_, *C*ON), 170.7 (C_q_, *C*ON), 168.9 (C_q_, *C*ON), 167.4 (C_q_, *C*ON), 139.2 (C_q_, *C*_ar_), 137.7 (C_q_, *C*_ar_), 137.4 (C_q_, *C*_ar_), 133.2 (C_q_, *C*_ar_), 130.7 (+, *C*_ar_H), 130.5 (+, *C*_ar_H), 130.4 (+, *C*_ar_H), 130.3 (+, *C*_ar_H), 129.7 (+, *C*_ar_H), 129.5 (+, *C*_ar_H), 129.3 (+, *C*_ar_H), 129.2 (+, *C*_ar_H), 129.1 (+, *C*_ar_H), 128.9 (+, 2 × *C*_ar_H), 128.8 (+, *C*_ar_H), 128.4 (+, *C*_ar_H), 127.1 (+, *C*_ar_H), 51.7 (–, CO*C*H_2_N), 51.6 (+, phe-*C*_α_H), 51.4 (–, CO*C*H_2_N), 48.9 (–, *N*1ph^p^Cl-*C*H_2_), 48.8 (–, *N*1ph-*C*H_2_), 47.7 (–, CO*C*H_2_N), 45.6 (–, *N*4am-*C*_1_H_2_), 40.2 (–, *N*4am-*C*_4_H_2_), 38.3 (–, phe-*C*H_2_), 24.9 (–, *N*4am-*C*_2_H_2_), 24.3 (–, *N*4am-*C*_3_H_2_). – **Analytical HPLC** (5–95% acetonitrile + 0.1% TFA in 30 min, detection at 218 nm): t_Ret_ = 11.2 min (>99%). – **MS**(m/z, MALDI-TOF): 604 [M+H]^+^, 626 [M+Na]^+^, 642 [M+K]^+^.

*cyclo*-(l‑Phe-*N*1ph^p^Cl-*N*4am(Rhod)-*N*1ph) (16-Rho)

Macrocycle **16** (15.1 mg, 25.0 µmol, 1.00 equiv.) was conjugated to rhodamine B following **GP8**. After purification *via* preparative reversed-phase HPLC (5–95% acetonitrile in water with 0.1% TFA in 35 min), the title compound was isolated as a pink solid (5.56 mg, 5.40 µmol, 22%).

**Analytical HPLC** (5–95% acetonitrile + 0.1% TFA in 30 min, detection at 218 nm): t_Ret_ = 14.6 min (89%). – **MS**(m/z, MALDI-TOF): 1029 [M+H]^+^.

*cyclo*-(l‑Phe-*N*3m-*N*1ph-*N*4am) (17)

Following **GP2**, the peptoid monomer *N*4am was immobilized using *N*-Boc-diaminobutane (**25**, 310 mg, 1.60 mmol, 8.00 equiv) for substitution. The subsequent peptoid monomers *N*1ph and *N*3m were incorporated following **GP4** using benzylamine (174 µL, 171 mg, 1.60 mmol, 8.00 equiv.) and *n-*butylamine (158 µL, 117 mg, 1.60 mmol, 8.00 equiv.) as submonomer, respectively. Subsequently, Fmoc-l‑phenylalanine (310 mg, 800 µmol, 4.00 equiv.) was coupled following **GP3**. Cleavage was performed by **GP5**. The crude linear precursor (42.9 mg, 65.6 µmol, 1.00 equiv.) was cyclized following **GP6** and deprotected according to **GP7**. After purification *via* preparative reversed-phase HPLC (5–95% acetonitrile in water with 0.1% TFA in 45 min), the macrocycle was isolated as a colorless solid (10.0 mg, 18.7 µmol, 9.6% over 11 steps).

The NMR spectra contain multiple signal sets. Chemical shifts of the most prominent isomer are given. **^1^H-NMR** (500 MHz, acetonitrile‑*d_3_*, ppm): δ = 7.41–7.16 (m, 10H, 5 × phe-C*H*, 5 × *N*1ph-C*H*), 6.57 (d, *J* = 10.2 Hz, 1H, phe-N_α_*H*), 5.27 (d, *J* = 14.5 Hz, 1H, COC*H*HN), 4.94–4.88 (m, 1H, phe-C_α_*H*), 4.47 (d, *J* = 16.9 Hz, 1H, COC*H*HN), 4.40 (d, *J* = 18.0 Hz, 1H, COC*H*HN), 4.00 (d, *J* = 18.1 Hz, 1H, *N*1ph-C*H*H), 3.90 (d, *J* = 17.1 Hz, 1H, COCH*H*N), 3.88–3.83 (m, 1H, *N*3m-C_1_*H*H), 3.64 (d, *J* = 18.4 Hz, 1H, COCH*H*N), 3.58 (d, *J* = 18.6 Hz, 1H, *N*1ph-CH*H*), 3.38–3.30 (m, 1H, *N*4am-C_1_*H*H), 3.30–3.23 (m, 1H, *N*4am-C_1_H*H*), 3.16 (dd, *J* = 13.7, 5.8 Hz, 1H, phe-C*H*H), 3.07 (d, *J* = 14.6 Hz, 1H, COCH*H*N), 3.00–2.93 (m, 1H, *N*4am-C_4_*H*H), 2.92–2.83 (m, 2H, *N*3m-C_1_H*H*, *N*4am-C_4_H*H*), 2.70 (dd, *J* = 13.7, 8.6 Hz, 1H, phe-CH*H*), 2.10–2.00 (m, 2H, *N*4am-N*H*_2_), 1.65–1.57 (m, 2H, *N*4am-C_2_*H*_2_), 1.55–1.36 (m, 4H, *N*3m-C_2_*H*_2_, *N*4am-C_3_*H*_2_), 1.32–1.18 (m, 2H, *N*3m-C_3_*H*_2_), 0.86 (t, *J* = 7.3 Hz, 3H, *N*3m-C*H*_3_).– **^13^C-NMR** (125 MHz, acetonitrile‑*d_3_*, ppm): δ = 170.6 (C_q_, *C*ON), 170.5 (C_q_, *C*ON), 169.4 (C_q_, *C*ON), 168.3 (C_q_, *C*ON), 139.5 (C_q_, *C*_ar_), 136.8 (C_q_, *C*_ar_), 130.6 (+, 2 × *C*_ar_H), 129.7 (+, 2 × *C*_ar_H), 128.9 (+, 2 × *C*_ar_H), 128.7 (+, *C*_ar_H), 128.3 (+, 2 × *C*_ar_H), 127.0 (+, *C*_ar_H), 52.0 (+, phe-*C*_α_H), 50.7 (–, CO*C*H_2_N), 50.2 (–, *N*1ph-*C*H_2_), 49.0 (–, CO*C*H_2_N), 48.8 (–, CO*C*H_2_N), 48.7 (–, *N*3m-*C*_1_H_2_), 48.4 (–, *N*4am-*C*_1_H_2_), 40.3 (–, *N*4am-*C*_4_H_2_), 38.5 (–, phe-*C*H_2_), 30.0 (–, *N*4am-*C*_2_H_2_), 24.9 (–, *N*4am-*C*_3_H_2_), 24.7 (–, *N*3m-*C*_2_H_2_), 20.6 (–, *N*3m-*C*_3_H_2_), 14.1 (+, *N*3m-*C*H_3_). – **Analytical HPLC** (5–95% acetonitrile + 0.1% TFA in 30 min, detection at 218 nm): 14.5 min (97%). – **MS**(m/z, MALDI-TOF): 536 [M+H]^+^, 558 [M+Na]^+^.

*cyclo*-(l‑Phe-*N*3m-*N*1ph-*N*4am(Rhod)) (17-Rhod)

Macrocycle **17** (4.82 mg, 9.00 µmol, 1.00 equiv.) was conjugated to rhodamine B following **GP8**. After purification *via* preparative reversed-phase HPLC (5–95% acetonitrile in water with 0.1% TFA in 35 min), the title compound was isolated as a pink solid (1.50 mg, 1.56 µmol, 17%).

**Analytical HPLC** (5–95% acetonitrile + 0.1% TFA in 30 min, detection at 218 nm): t_Ret_ = 18.6 min (>99%). – **MS**(m/z, MALDI-TOF): 961 [M+H]^+^.

*cyclo*-(l‑Phe-*N*4am-*N*1ph-l-Tyr) (18)

*O*‑*^tert^*butyl-Fmoc-l‑tyrosine (368 mg, 800 µmol, 4.00 equiv.) was immobilized by **GP1**. The subsequent peptoid monomers *N*4am and *N*1ph were incorporated following **GP4** using *N*-Boc-diaminobutane (**25**, 310 mg, 1.60 mmol, 8.00 equiv) and benzylamine (174 µL, 171 mg, 1.60 mmol, 8.00 equiv.) as submonomer, respectively. Subsequently, Fmoc-l‑phenylalanine (310 mg, 800 µmol, 4.00 equiv.) was coupled following **GP3**. Cleavage was performed by **GP5**. The crude linear precursor (76.0 mg, 100 µmol, 1.00 equiv.) was cyclized following **GP6** and deprotected according to **GP7**. After purification *via* preparative reversed-phase HPLC (5–95% acetonitrile in water with 0.1% TFA in 45 min), the macrocycle was isolated as a colorless solid (16.4 mg, 28.0 µmol, 28% over 11 steps).

The NMR spectra contain multiple signal sets. Chemical shifts of the most prominent isomer are given. **^1^H-NMR** (500 MHz, DMSO‑*d_6_*, ppm): δ = 7.96 (d, *J* = 10.3 Hz, 1H, phe-N_α_*H*), 7.40–7.18 (m, 10H, 5 × phe-C_ar_*H*, 5 × *N*1ph-C_ar_*H*), 6.91 (d, *J* = 8.5 Hz, 2H, 2 × tyr-C_ar_*H*), 6.64 (d, *J* = 8.5 Hz, 1H, 2 × tyr-C_ar_*H*), 6.26 (d, *J* = 8.4 Hz, 1H, tyr-N_α_*H*), 4.96 (td, *J* = 10.3, 4.7 Hz, 1H, phe-C_α_*H*), 4.49 (d, *J* = 16.6 Hz, 1H, COC*H*HN), 4.36 (d, *J* = 17.7 Hz, 1H, *N*1ph-C*H*H), 4.31 (d, *J* = 16.5 Hz, 1H, COCH*H*N), 3.88–3.79 (m, 2H, tyr-C_α_*H*, *N*4am-C_1_*H*H), 3.77 (d, *J* = 18.0 Hz, 1H, *N*1ph-CH*H*), 3.61 (d, *J* = 15.1 Hz, 1H, COC*H*HN), 3.55–3.44 (m, 1H, COCH*H*N), 3.13 (dd, *J* = 13.8, 4.7 Hz, 1H, phe-C*H*H), 2.88–2.72 (m, 5H, phe-CH*H*, tyr-C*H*H, *N*4am-C_1_H*H*, *N*4am-C_4_*H*_2_), 2.27–2.21 (m, 1H, tyr-C*H*H), 1.55–1.41 (m, 6H, *N*4am-C_2_*H*_2_, *N*4am-C_3_*H*_2_, *N*4am-N*H*_2_). – **^13^C-NMR** (125 MHz, DMSO‑*d_6_*, ppm): δ = 170.6 (C_q_, *C*ON), 170.1 (C_q_, 2 × *C*ON), 169.6 (C_q_, *C*ON), 156.30 (C_q_, tyr-*C*_ar_O), 137.9 (C_q_, *C*_ar_), 136.6 (C_q_, *C*_ar_), 130.1 (C_q_, *C*_ar_), 130.0 (+, 2 × tyr-*C*_ar_H), 129.7 (+, 2 × *C*_ar_H), 128.8 (+, 2 × *C*_ar_H), 127.7 (+, 2 × *C*_ar_H), 127.2 (+, 2 × *C*_ar_H), 126.0 (+, 2 × *C*_ar_H), 115.1 (+, 2 × tyr-*C*_ar_H), 58.6 (+, tyr-*C*_α_H), 52.9 (–, CO*C*H_2_N), 52.1 (–, CO*C*H_2_N), 50.2 (+, phe-*C*_α_H), 47.9 (–, *N*1ph-*C*H_2_), 47.8 (–, *N*4am-*C*_1_H_2_), 38.7 (–, *N*4am-*C*_4_H_2_), 37.7 (–, tyr-*C*H_2_), 37.4 (–, phe-*C*H_2_), 24.4 (–, *N*4am-*C*_2_H_2_), 23.8 (–, *N*4am-*C*_3_H_2_). – **Analytical HPLC** (5–95% acetonitrile + 0.1% TFA in 30 min, detection at 218 nm): t_Ret_ = 10.4 min (94%). – **MS**(m/z, MALDI-TOF): 586 [M+H]^+^, 608 [M+Na]^+^, 624 [M+K]^+^.

*cyclo*-(l‑Phe-*N*4am(Rhod)-*N*1ph-l-Tyr) (18-Rhod)

Macrocycle **18** (5.27 mg, 9.00 µmol, 1.00 equiv.) was conjugated to rhodamine B following **GP8**. After purification *via* preparative reversed-phase HPLC (5–95% acetonitrile in water with 0.1% TFA in 35 min), the title compound was isolated as a pink solid (0.60 mg, 0.593 µmol, 7.0%).

**Analytical HPLC** (5–95% acetonitrile + 0.1% TFA in 30 min, detection at 218 nm): t_Ret_ = 15.8 min (93%). – **MS**(m/z, MALDI-TOF): 1011 [M+H]^+^, 1033 [M+Na]^+^, 1049 [M+K]^+^.

*cyclo*-(l‑Trp-*N*1ph-*N*1ph-l-Lys) (19)

*N*-Boc-Fmoc-l‑lysine (375 mg, 800 µmol, 4.00 equiv.) was immobilized by **GP1**. The subsequent peptoid monomers *N*1ph were incorporated following **GP4** using benzylamine (174 µL, 171 mg, 1.60 mmol, 8.00 equiv.) as a submonomer. Subsequently, Fmoc-l‑tryptophan (341 mg, 800 µmol, 4.00 equiv.) was coupled following **GP3**. Cleavage was performed by **GP5**. The crude linear precursor (72.7 mg, 100 µmol, 1.00 equiv.) was cyclized following **GP6** and deprotected according to **GP7**. After purification *via* preparative reversed-phase HPLC (5–95% acetonitrile in water with 0.1% TFA in 45 min), the macrocycle was isolated as a colorless solid (17.3 mg, 28.4 µmol, 17% over 11 steps).

The NMR spectra contain multiple signal sets. Chemical shifts of the most prominent isomer are given. **^1^H-NMR** (500 MHz, methanol‑*d_4_*, ppm): δ = 7.63 (dt, *J* = 7.8, 1.0 Hz, 1H, trp-C*H*), 7.39–7.16 (m, 13H, lys-N_α_*H*, trp-C*H*, trp-N_α_*H*, 10 × *N*1ph-C*H*), 7.15–7.00 (m, 4H, 3 × trp-C*H*, trp-N*H*), 5.50 (d, *J* = 14.8 Hz, 1H, COC*H*HN), 5.32 (dd, *J* = 10.2, 5.1 Hz, 1H, trp-C_α_*H*), 4.57 (d, *J* = 16.4 Hz, 1H, COC*H*HN), 4.40 (d, *J* = 18.1 Hz, 1H, *N*1ph-C*H*H), 4.17 (d, *J* = 16.6 Hz, 1H, COCH*H*N), 4.01 (d, *J* = 14.7 Hz, 1H, COCH*H*N), 3.88 (d, *J* = 15.4 Hz, 1H, *N*1ph-C*H*H), 3.74 (d, *J* = 15.4 Hz, 1H, *N*1ph-CH*H*), 3.67–3.61 (m, 1H, lys-C_α_*H*), 3.56 (d, *J* = 17.9 Hz, 1H, *N*1ph-CH*H*), 3.40–3.35 (m, 1H, trp-C*H*H), 3.20–3.11 (m, 1H, trp-CH*H*), 2.73 (dt, *J* = 12.5, 7.5 Hz, 1H, lys-C_ε_*H*H), 2.63 (dt, *J* = 12.5, 7.7 Hz, 1H, lys-C_ε_H*H*), 1.70–1.57 (m, 4H, lys-C_γ_*H*_2_, lys-N*H*_2_), 1.43–1.36 (m, 2H, lys-C_δ_*H*_2_), 1.08–0.99 (m, 2H, lys-C_β_*H*_2_). – **^13^C-NMR** (125 MHz, methanol‑*d_4_*, ppm): δ = 172.5 (C_q_, *C*ON), 171.4 (C_q_, *C*ON), 171.3 (C_q_, *C*ON), 171.1 (C_q_, *C*ON), 136.4 (C_q_, trp-*C*_ar_), 136.3 (C_q_, *N*1ph-*C*_ar_), 135.8 (C_q_, *N*1ph-*C*_ar_), 128.7 (+, 2 × *N*1ph-*C*_ar_H), 128.4 (+, 4 × *N*1ph-*C*_ar_H), 128.2 (+, 2 × *N*1ph-*C*_ar_H), 127.6 (+, *N*1ph-*C*_ar_H), 127.3 (+, *N*1ph-*C*_ar_H), 126.5 (+, trp-*C*_ar_H), 123.8 (C_q_, trp-*C*_ar_), 120.8 (+, trp-*C*_ar_H), 118.4 (+, trp-*C*_ar_H), 118.3 (+, trp-*C*_ar_H), 110.7 (+, trp-*C*_ar_H), 110.0 (C_q_, trp-*C*_ar_), 57.9 (+, lys-*C*_α_H), 53.3 (–, *N*1ph-*C*H_2_), 52.9 (–, CO*C*H_2_N), 51.2 (–, CO*C*H_2_N), 50.5 (+, trp-*C*_α_H), 46.5 (–, *N*1ph-*C*H_2_), 39.0 (–, lys-*C*_ε_H_2_), 32.0 (–, lys-*C*_β_H_2_), 26.8 (–, lys-*C*_δ_H_2_), 26.5 (–, trp-*C*H_2_), 22.4 (–, lys-*C*_γ_H_2_). – **Analytical HPLC** (5–95% acetonitrile + 0.1% TFA in 30 min, detection at 218 nm): t_Ret_ = 12.0 min (21%) and 12.2 min (70%). – **MS**(m/z, MALDI-TOF): 609 [M+H]^+^, 631 [M+Na]^+^, 647 [M+K]^+^.

*cyclo*-(l‑Trp-*N*1ph-*N*1ph-l-Lys(Rhod)) (19-Rhod)

Macrocycle **19** (5.00 mg, 8.21 µmol, 1.00 equiv.) was conjugated to rhodamine B following **GP8**. After purification *via* preparative reversed-phase HPLC (5–95% acetonitrile in water with 0.1% TFA in 35 min), the title compound was isolated as a pink solid (3.20 mg, 3.10 µmol, 38%).

**Analytical HPLC** (5–95% acetonitrile + 0.1% TFA in 30 min, detection at 218 nm): t_Ret_ = 15.2 min (>99%). – **MS**(m/z, MALDI-TOF): 1034 [M+H]^+^, 1056 [M+Na]^+^.

*cyclo*-(l‑Trp-*N*1ph^p^Cl-*N*1ph^p^Cl-l-Lys) (20)

*N*-Boc-Fmoc-l‑lysine (375 mg, 800 µmol, 4.00 equiv.) was immobilized by **GP1**. The subsequent peptoid monomers *N*1ph^p^Cl were incorporated following **GP4** using *p*‑chlorobenzylamine (195 µL, 227 mg, 1.60 mmol, 8.00 equiv.) as a submonomer. Subsequently, Fmoc-l‑tryptophan (341 mg, 800 µmol, 4.00 equiv.) was coupled following **GP3**. Cleavage was performed by **GP5**. The crude linear precursor (79.4 mg, 100 µmol, 1.00 equiv.) was cyclized following **GP6** and deprotected according to **GP7**. After purification *via* preparative reversed-phase HPLC (5–95% acetonitrile in water with 0.1% TFA in 45 min), the macrocycle was isolated as a colorless solid (7.50 mg, 10.8 µmol, 8.5% over 11 steps).

The NMR spectra contain multiple signal sets. Chemical shifts of the most prominent isomer are given. **^1^H-NMR** (500 MHz, methanol‑*d_4_*, ppm): δ = 7.53 (dt, *J* = 7.9, 1.0 Hz, 1H, trp-C*H*), 7.27–7.16 (m, 7H, lys-N_α_*H*, trp-C*H*, trp-N_α_*H*, 4 × *N*1ph^p^Cl-C*H*), 7.11–7.06 (m, 2H, 2 × *N*1ph^p^Cl-C*H*), 7.02–6.90 (m, 6H, 3 × trp-C*H*, trp-N*H*, 2 × *N*1ph^p^Cl-C*H*), 5.29 (d, *J* = 15.0 Hz, 1H, COC*H*HN), 5.22 (dd, *J*= 10.1, 5.1 Hz, 1H, trp-C_α_*H*), 4.46 (d, *J* = 16.7 Hz, 1H, COC*H*HN), 4.32 (d, *J* = 18.0 Hz, 1H, *N*1ph^p^Cl-C*H*H), 4.08 (d, *J* = 16.7 Hz, 1H, COCH*H*N), 3.94 (d, *J* = 14.9 Hz, 1H, COCH*H*N), 3.78 (d, *J* = 15.3 Hz, 1H, *N*1ph^p^Cl-C*H*H), 3.63 (d, *J* = 15.4 Hz, 1H, *N*1ph^p^Cl-CH*H*), 3.57–3.48 (m, 1H, lys-C_α_*H*), 3.44 (d, *J* = 17.9 Hz, 1H, *N*1ph^p^Cl-CH*H*), 3.30–3.23 (m, 1H, trp-C*H*H), 3.02 (dd, *J* = 14.8, 10.2 Hz, 1H, trp-CH*H*), 2.62 (dt, *J* = 14.9, 7.5 Hz, 1H, lys-C_ε_*H*H), 2.53 (dt, *J* = 14.8, 7.4 Hz, 1H, lys-C_ε_H*H*), 1.61–1.48 (m, 2H, lys-N*H*_2_), 1.31–1.25 (m, 2H, lys-C_δ_*H*_2_), 0.98–0.90 (m, 3H, lys-C_β_*H*_2_, lys-C_γ_*H*H), 0.84–0.75 (m, 1H, lys-C_γ_H*H*). – **^13^C-NMR** (125 MHz, methanol‑*d_4_*, ppm): δ = 172.4 (C_q_, *C*ON), 171.3 (C_q_, *C*ON), 171.2 (C_q_, *C*ON), 171.1 (C_q_, *C*ON), 136.4 (C_q_, trp-*C*_ar_), 135.3 (C_q_, *N*1ph^p^Cl-*C*_ar_), 134.7 (C_q_, *N*1ph^p^Cl-*C*_ar_), 133.5 (C_q_, *N*1ph^p^Cl-*C*_ar_), 133.1 (C_q_, *N*1ph^p^Cl-*C*_ar_), 129.8 (+, 2 × *N*1ph^p^Cl-*C*_ar_H), 128.8 (+, 2 × *N*1ph^p^Cl-*C*_ar_H), 128.3 (+, 2 × *N*1ph^p^Cl-*C*_ar_H), 128.2 (+, 2 × *N*1ph^p^Cl-*C*_ar_H), 128.1 (C_q_, trp-*C*_ar_), 123.8 (+, trp-*C*_ar_H), 120.8 (+, trp-*C*_ar_H), 118.4 (+, trp-*C*_ar_H), 118.3 (+, trp-*C*_ar_H), 110.7 (+, trp-*C*_ar_H), 110.0 (C_q_, trp-*C*_ar_), 57.9 (+, lys-*C*_α_H), 53.3 (–, *N*1ph^p^Cl-*C*H_2_), 52.9 (–, CO*C*H_2_N), 50.7 (–, CO*C*H_2_N), 50.5 (+, trp-*C*_α_H), 46.9 (–, *N*1ph^p^Cl-*C*H_2_), 39.0 (–, lys-*C*_ε_H_2_), 32.0 (–, lys-*C*_β_H_2_), 26.8 (–, lys-*C*_δ_H_2_), 26.6 (–, trp-*C*H_2_), 22.4 (–, lys-*C*_γ_H_2_). – **Analytical HPLC** (5–95% acetonitrile + 0.1% TFA in 30 min, detection at 218 nm): t_Ret_ = 13.8 min (88%). – **MS**(m/z, MALDI-TOF): 677 [M+H]^+^, 699 [M+Na]^+^, 715 [M+K]^+^.

*cyclo*-(l‑Trp-*N*1ph^p^Cl-*N*1ph^p^Cl-l-Lys(Rhod)) (20-Rhod)

Macrocycle **20** (5.00 mg, 7.38 µmol, 1.00 equiv.) was conjugated to rhodamine B following **GP8**. After purification *via* preparative reversed-phase HPLC (5–95% acetonitrile in water with 0.1% TFA in 35 min), the title compound was isolated as a pink solid (1.70 mg, 1.54 µmol, 21%).

**Analytical HPLC** (5–95% acetonitrile + 0.1% TFA in 30 min, detection at 218 nm): t_Ret_ = 16.6 min (91%). – **MS**(m/z, MALDI-TOF): 1102 [M+H]^+^, 1124 [M+Na]^+^, 1140 [M+K]^+^.

*cyclo*-(l‑Tyr-*N*3m-*N*3m-l-Lys) (21)

*N*-Boc-Fmoc-l‑lysine (375 mg, 800 µmol, 4.00 equiv.) was immobilized by **GP1**. The subsequent peptoid monomers *N*3m were incorporated following **GP4** using *n‑*butylamine (158 µL, 117 mg, 1.60 mmol, 8.00 equiv.) as a submonomer. Subsequently, *O*‑*^tert^*butyl-Fmoc-l‑tyrosine (368 mg, 800 µmol, 4.00 equiv.) was coupled following **GP3**. Cleavage was performed by **GP5**. The crude linear precursor (69.2 mg, 100 µmol, 1.00 equiv.) was cyclized following **GP6** and deprotected according to **GP7**. After purification *via* preparative reversed-phase HPLC (5–95% acetonitrile in water with 0.1% TFA in 45 min), the macrocycle was isolated as a colorless solid (14.8 mg, 28.6 µmol, 13% over 11 steps).

The NMR spectra contain multiple signal sets. Chemical shifts of the most prominent isomer are given. **^1^H-NMR** (500 MHz, methanol‑*d_4_*, ppm): δ = 7.02 (d, *J* = 8.3 Hz, 2H, 2 × tyr-C*H*), 6.73–6.68 (m, 2H, lys-N_α_*H*, tyr-N_α_*H*), 6.65 (d, *J* = 8.6 Hz, 2H, 2 × tyr-C*H*), 5.04 (dd, *J* = 11.2, 4.6 Hz, 1H, tyr-C_α_*H*), 4.33 (d, *J* = 17.7 Hz, 1H, COC*H*HN), 4.05–3.96 (m, 1H, *N*3m-C_1_*H*H) 3.91 (d, *J* = 15.2 Hz, 1H, COC*H*HN), 3.83 (d, *J*= 17.7 Hz, 1H, COCH*H*N), 3.75 (d, *J* = 15.4 Hz, 1H, COCH*H*N), 3.56–3.48 (m, 1H, lys-C_α_*H*), 3.38–3.33 (m, 1H, *N*3m-C_1_*H*H),3.12 (dd, *J* = 14.4, 4.6 Hz, 1H, tyr-C*H*H), 2.88–2.82 (m, 4H, lys-C_ε_*H*_2_, 2 × *N*3m-C_1_H*H*), 2.76–2.69 (m, 1H, tyr-CH*H*), 1.70–1.49 (m, 10H, lys-C_γ_*H*_2_, lys-C_δ_*H*_2_, lys-N*H*_2_, 2 × *N*3m-C_2_*H*_2_), 1.41–1.24 (m, 6H, lys-C_β_*H*_2_, 2 × *N*3m-C_3_*H*_2_), 0.99–0.92 (m, 6H, 2 × *N*3m-C*H*_3_). – **^13^C-NMR** (125 MHz, methanol‑*d_4_*, ppm): δ = 172.7 (C_q_, *C*ON), 170.9 (C_q_, *C*ON), 170.8 (C_q_, *C*ON), 170.7 (C_q_, *C*ON), 155.4 (C_q_, tyr-*C*_ar_O), 130.8 (+, 2 × tyr-*C*_ar_H), 129.5 (C_q_, tyr-*C*_ar_),114.4 (+, 2 × tyr-*C*_ar_H), 57.9 (+, lys-*C*_α_H), 52.2 (–, CO*C*H_2_N), 50.7 (+, trp-*C*_α_H), 49.3 (–, *N*3m-*C*_1_H_2_), 49.2 (–, *N*3m-*C*_1_H_2_), 48.2 (–, CO*C*H_2_N), 39.1 (–, lys-*C*_ε_H_2_), 36.4 (–, trp-*C*H_2_), 32.8 (–, lys-*C*_β_H_2_), 30.5 (–, lys-*C*_γ_H_2_), 30.0 (–, lys-*C*_δ_H_2_), 29.0 (–, *N*3m-*C*_2_H_2_), 26.8 (–, *N*3m-*C*_2_H_2_), 19.8 (–, *N*3m-*C*_3_H_2_), 19.5 (–, *N*3m-*C*_3_H_2_), 12.9 (+, *N*3m-*C*H_3_), 12.7 (+, *N*3m-*C*H_3_). – **Analytical HPLC** (5–95% acetonitrile + 0.1% TFA in 30 min, detection at 218 nm): t_Ret_ = 9.99 min (91%). – **MS**(m/z, MALDI-TOF): 518 [M+H]^+^, 540 [M+Na]^+^.

*cyclo*-(l‑Tyr-*N*3m-*N*3m-l-Lys(Rhod)) (21-Rhod)

Macrocycle **21** (5.00 mg, 18.5 µmol, 1.00 equiv.) was conjugated to rhodamine B following **GP8**. After purification *via* preparative reversed-phase HPLC (5–95% acetonitrile in water with 0.1% TFA in 35 min), the title compound was isolated as a pink solid (1.00 mg, 1.06 µmol,6 %).

**Analytical HPLC** (5–95% acetonitrile + 0.1% TFA in 30 min, detection at 218 nm): t_Ret_ = 15.2 min (>99%). – **MS**(m/z, MALDI-TOF): 943 [M+H]^+^.

*cyclo*-(l‑Phe-*N*1ph-*N*1ph-*N*1ph-*N*4am-l‑Phe) (22)

Fmoc-l‑phenylalanine (310 mg, 800 µmol, 4.00 equiv.) was immobilized by **GP1**. The subsequent peptoid monomers *N*1ph and *N*4am were incorporated following **GP4** using benzylamine (174 µL, 171 mg, 1.60 mmol, 8.00 equiv.) and *N*-Boc-diaminobutane (**25**, 310 mg, 1.60 mmol, 8.00 equiv) as submonomer, respectively. Subsequently, Fmoc-l‑phenylalanine (310 mg, 800 µmol, 4.00 equiv.) was coupled following **GP3**. Cleavage was performed by **GP5**. The crude linear precursor (130 mg, 130 µmol, 1.00 equiv.) was cyclized following **GP6** and deprotected according to **GP7**. After purification *via* preparative reversed-phase HPLC (5–95% acetonitrile in water with 0.1% TFA in 45 min), the macrocycle was isolated as a colorless solid (25.2 mg, 29.2 µmol, 15% over 15 steps).

The NMR spectra contain multiple signal sets. Chemical shifts of the most prominent isomer are given. **^1^H-NMR** (500 MHz, acetonitrile‑*d_3_*, ppm): δ = 8.71 (d, *J* = 6.7 Hz, 1H, phe-N_α_*H*), 7.36–7.08 (m, 26H, 8 × phe-C*H*, phe-N_α_*H*, 15 × *N*1ph-C*H*), 6.80–6.71 (m, 2H, 2 × phe-C*H*), 5.38 (d, *J* = 15.3 Hz, 1H, COC*H*HN), 5.19 (d, *J* = 15.1 Hz, 1H, COC*H*HN), 4.68–4.50 (m, 2H, phe-C_α_*H*, COC*H*HN), 4.34–4.12 (m, 6H, phe-C_α_*H*, COC*H*HN, COCH*H*N, *N*4am-C_1_*H*H, 2 × *N*1ph-C*H*H), 4.00 (d, *J* = 18.2 Hz, 1H, *N*1ph-C*H*H), 3.93–3.71 (m, 5H, 2 × COCH*H*N, 3 × *N*1ph-CH*H*), 3.31 (d, *J* = 16.6 Hz, 1H, COCH*H*N), 3.23–3.12 (m, 1H, phe-C*H*H), 3.08–3.01 (m, 2H, phe-C*H*H, *N*4am-C_4_*H*H), 2.99–2.82 (m, 5H, 2 × phe-CH*H*, *N*4am-C_4_H*H*, *N*4am-N*H*_2_), 2.38 (d, *J* = 13.7 Hz, 1H, *N*4am-C_1_H*H*), 1.90–1.73 (m, 2H, *N*4am-C_2_*H*_2_), 1.63–1.47 (m, 2H, *N*4am-C_3_*H*_2_). – **^13^C-NMR** (125 MHz, acetonitrile‑*d_3_*, ppm): δ = 173.2 (C_q_, *C*ON), 172.3 (C_q_, *C*ON), 171.0 (C_q_, *C*ON), 170.9 (C_q_, *C*ON), 170.7 (C_q_, *C*ON), 169.3 (C_q_, *C*ON), 139.8 (C_q_, *C*_ar_), 138.5 (C_q_, *C*_ar_), 138.0 (C_q_, *C*_ar_), 137.0 (C_q_, *C*_ar_), 136.6 (C_q_, *C*_ar_), 130.5 (+,*C*_ar_H), 130.3 (+, 2 × *C*_ar_H), 130.2 (+, *C*_ar_H), 129.7 (+, *C*_ar_H), 129.5 (+, 2 × *C*_ar_H), 129.4 (+, 5 × *C*_ar_H), 129.3 (+, 3 × *C*_ar_H), 129.2 (+, 2 × *C*_ar_H), 129.0 (+, *C*_ar_H), 128.9 (+, *C*_ar_H), 128.3 (+, *C*_ar_H), 128.2 (+, *C*_ar_H), 128.1 (+, *C*_ar_H), 127.9 (+, *C*_ar_H), 127.7 (+, *C*_ar_H), 127.0 (+, *C*_ar_H), 57.2 (+, phe-*C*_α_H), 55.6 (+, phe-*C*_α_H), 53.0 (–, CO*C*H_2_N), 51.6 (–, CO*C*H_2_N), 51.1 (–, CO*C*H_2_N), 51.0 (–, CO*C*H_2_N), 49.0 (–, *N*1ph-*C*H_2_), 48.8 (–, *N*1ph-*C*H_2_), 47.4 (–, *N*1ph-*C*H_2_), 43.9 (–, *N*4am-*C*_1_H_2_), 40.2 (–, *N*4am-*C*_4_H_2_), 37.9 (–, phe-*C*H_2_), 37.1 (–, phe-*C*H_2_), 24.0 (–, *N*4am-*C*_2_H_2_), 22.9 (–, *N*4am-*C*_3_H_2_).– **Analytical HPLC** (5–95% acetonitrile + 0.1% TFA in 30 min, detection at 218 nm): t_Ret_ = 11.1 min (87%). – **MS**(m/z, MALDI-TOF): 864 [M+H]^+^, 886 [M+Na]^+^, 902 [M+K]^+^.

*cyclo*-(l‑Phe-*N*1ph-*N*1ph-*N*1ph-*N*4am(Rhod)-l‑Phe) (22-Rhod)

Macrocycle **22** (15.0 mg, 17.4 µmol, 1.00 equiv.) was conjugated to rhodamine B following **GP8**. After purification *via* preparative reversed-phase HPLC (5–95% acetonitrile in water with 0.1% TFA in 35 min), the title compound was isolated as a pink solid (13.5 mg, 15.9 µmol, 91%).

**Analytical HPLC** (5–95% acetonitrile + 0.1% TFA in 30 min, detection at 218 nm): t_Ret_ = 17.6 min (>99%). – **MS**(m/z, MALDI-TOF): 1289 [M+H]^+^, 1311 [M+Na]^+^, 1327 [M+K]^+^.

*cyclo*-(l‑Phe-*N*1ph^p^Cl-*N*1ph^p^Cl-*N*1ph^p^Cl-*N*4am-l‑Phe) (23)

Fmoc-l‑phenylalanine (310 mg, 800 µmol, 4.00 equiv.) was immobilized by **GP1**. The subsequent peptoid monomers *N*1ph^p^Cl and *N*4am were incorporated following **GP4** using *p*-chlorobenzylamine (195 µL, 227 mg, 1.60 mmol, 8.00 equiv.) and *N*-Boc-diaminobutane (**25**, 310 mg, 1.60 mmol, 8.00 equiv.) as submonomer, respectively. Subsequently, Fmoc-l‑phenylalanine (310 mg, 800 µmol, 4.00 equiv.) was coupled following **GP3**. Cleavage was performed by **GP5**. The crude linear precursor (140 mg, 130 µmol, 1.00 equiv.) was cyclized following **GP6** and deprotected according to **GP7**. After purification *via* preparative reversed-phase HPLC (5–95% acetonitrile in water with 0.1% TFA in 45 min), the macrocycle was isolated as a colorless solid (46.3 mg, 47.9 µmol, 12% over 15 steps).

The NMR spectra contain multiple signal sets. Chemical shifts of the most prominent isomer are given. **^1^H-NMR** (500 MHz, acetonitrile‑*d_3_*, ppm): δ = 8.64 (d, *J* = 6.7 Hz, 1H, phe-N_α_*H*), 7.54–6.99 (m, 23H, 8 × phe-C*H*, phe-N_α_*H*, 12 × *N*1ph^p^Cl-C*H*), 6.78–6.68 (m, 1H, 2 × phe-C*H*), 5.26 (d, *J* = 15.5 Hz, 1H, COC*H*HN), 5.09 (d, *J* = 15.2 Hz, 1H, COC*H*HN), 4.68–4.55 (m, 2H, phe-C_α_*H*, COC*H*HN), 4.40–4.09 (m, 6H, phe-C_α_*H*, COC*H*HN, COCH*H*N, *N*4am-C_1_*H*H, 2 × *N*1ph^p^Cl-C*H*H), 4.03–3.92 (m, 1H, *N*1ph^p^Cl-C*H*H), 3.92–3.71 (m, 5H, 2 × COCH*H*N, 3 × *N*1ph^p^Cl-CH*H*), 3.33 (d, *J* = 16.6 Hz, 1H, COCH*H*N), 3.21–2.99 (m, 3H, 2 × phe-C*H*H, *N*4am-C_4_*H*H), 2.98–2.79 (m, 5H, 2 × phe-CH*H*, *N*4am-C_4_H*H*, *N*4am-N*H*_2_), 2.37 (d, *J* = 13.9 Hz, 1H, *N*4am-C_1_H*H*), 1.86–1.70 (m, 2H, *N*4am-C_2_*H*_2_), 1.60–1.46 (m, 2H, *N*4am-C_3_*H*_2_). – **^13^C-NMR** (125 MHz, acetonitrile‑*d_3_*, ppm): δ = 173.2 (C_q_, *C*ON), 172.2 (C_q_, *C*ON), 171.0 (C_q_, *C*ON), 170.8 (C_q_, *C*ON), 169.3 (C_q_, *C*ON), 163.3 (C_q_, *C*ON), 139.7 (C_q_, *C*_ar_), 138.1 (C_q_, *C*_ar_), 137.9 (C_q_, *C*_ar_), 137.5 (C_q_, *C*_ar_), 136.9 (C_q_, *C*_ar_), 136.1 (C_q_, *C*_ar_), 133.9 (C_q_, *C*_ar_), 133.5 (C_q_, *C*_ar_), 130.9 (+, 2 × *C*_ar_H), 130.7 (+, *C*_ar_H), 130.5 (+, *C*_ar_H), 130.2 (+, *C*_ar_H), 129.8 (+, *C*_ar_H), 129.6 (+, *C*_ar_H), 129.5 (+, 3 × *C*_ar_H), 129.4 (+, 2 × *C*_ar_H), 129.3 (+, 2 × *C*_ar_H), 129.2 (+, 3 × *C*_ar_H), 129.0 (+, 2 × *C*_ar_H), 127.9 (+, *C*_ar_H), 127.7 (+, *C*_ar_H), 127.0 (+, *C*_ar_H), 57.1 (+, phe-*C*_α_H), 55.5 (+, phe-*C*_α_H), 52.4 (–, CO*C*H_2_N), 51.2 (–, CO*C*H_2_N), 51.0 (–, CO*C*H_2_N), 50.6 (–, CO*C*H_2_N), 49.1 (–, *N*1ph^p^Cl-*C*H_2_), 49.0 (–, *N*1ph^p^Cl-*C*H_2_), 47.5 (–, *N*1ph^p^Cl-*C*H_2_), 43.9 (–, *N*4am-*C*_1_H_2_), 40.1 (–, *N*4am-*C*_4_H_2_), 37.1 (–, phe-*C*H_2_), 37.0 (–, phe-*C*H_2_), 24.0 (–, *N*4am-*C*_2_H_2_), 22.9 (–, *N*4am-*C*_3_H_2_). – **Analytical HPLC** (5–95% acetonitrile + 0.1% TFA in 30 min, detection at 218 nm): t_Ret_ = 16.2 min (83%). – **MS**(m/z, MALDI-TOF): 966 [M+H]^+^, 988 [M+Na]^+^.

*cyclo*-(l‑Phe-*N*1ph^p^Cl-*N*1ph^p^Cl-*N*1ph^p^Cl-*N*4am(Rhod)-l‑Phe) (23-Rhod)

Macrocycle **23** (20.3 mg, 21.0 µmol, 1.00 equiv.) was conjugated to rhodamine B following **GP8**. After purification *via* preparative reversed-phase HPLC (5–95% acetonitrile in water with 0.1% TFA in 35 min), the title compound was isolated as a pink solid (17.9 mg, 12.9 µmol, 61%).

**Analytical HPLC** (5–95% acetonitrile + 0.1% TFA in 30 min, detection at 218 nm): t_Ret_ = 19.5 min (>99%). – **MS**(m/z, MALDI-TOF): 1391 [M+H]^+^, 1413 [M+Na]^+^, 1429 [M+K]^+^.

*cyclo*-(l‑Phe-*N*3m-*N*3m-*N*1ph-*N*4am-l‑Phe) (24)

Fmoc-l‑phenylalanine (310 mg, 800 µmol, 4.00 equiv.) was immobilized by **GP1**. The subsequent peptoid monomers *N*3m, *N*1ph and *N*4am were incorporated following **GP4** using *n-*butylamine (158 µL, 117 mg, 1.60 mmol, 8.00 equiv.), benzylamine (174 µL, 171 mg, 1.60 mmol, 8.00 equiv.) and *N*-Boc-diaminobutane (**25**, 310 mg, 1.60 mmol, 8.00 equiv.) as submonomer, respectively. Subsequently, Fmoc-l‑phenylalanine (310 mg, 800 µmol, 4.00 equiv.) was coupled following **GP3**. Cleavage was performed by **GP5**. The crude linear precursor (86.9 mg, 95.1 µmol, 1.00 equiv.) was cyclized following **GP6** and deprotected according to **GP7**. After purification *via* preparative reversed-phase HPLC (5–95% acetonitrile in water with 0.1% TFA in 45 min), the macrocycle was isolated as a colorless solid (15.3 mg, 17.0 µmol, 9.6% over 15 steps).

The NMR spectra contain multiple signal sets. Chemical shifts of the most prominent isomer are given. **^1^H-NMR** (500 MHz, acetonitrile‑*d_3_*, ppm): δ = 8.88 (d, *J* = 6.4 Hz, 1H, phe-N_α_*H*), 7.41–7.12 (m, 14H, 13 × C_ar_*H*, phe-N_α_*H*), 6.81–6.76 (m, 2H, 2 × C_ar_*H*), 5.35 (d, *J* = 15.3 Hz, 1H, COC*H*HN), 4.30 (d, *J* = 16.7 Hz, 1H, COC*H*HN), 4.24 (d, *J* = 18.4 Hz, 1H, COC*H*HN), 4.19–4.07 (m, 3H, phe-C_α_*H*, *N*1ph-C*H*H, *N*4am-C_1_*H*H), 3.99 (d, *J* = 18.5 Hz, 1H, COCH*H*N), 3.87 (d, *J* = 17.8 Hz, 1H, COC*H*HN), 3.83–3.75 (m, 2H, phe-C_α_*H*, *N*1ph-CH*H*), 3.74–3.66 (m, 3H, 2 × COCH*H*N, *N*3m-C_1_*H*H), 3.43 (d, *J* = 16.6 Hz, 1H, COCH*H*N), 3.29–3.20 (m, 1H, *N*3m-C_1_*H*H), 3.17–3.10 (m, 2H, phe-C*H*H, *N*3m-C_1_H*H*), 3.08–3.00 (m, 2H, phe-C*H*H, *N*4am-C_4_*H*H), 2.94–2.84 (m, 2H, phe-CH*H*, *N*3m-C_1_H*H*), 2.83–2.75 (m, 1H, *N*4am-C_4_H*H*), 2.45 (dd, *J* = 13.9, 8.3 Hz, 1H, phe-CH*H*), 2.32 (dt, *J* = 13.7, 3.7 Hz, 1H, *N*4am-C_1_H*H*), 1.83–1.70 (m, 2H, 2 × *N*4am-C*H*H), 1.65–1.54 (m, 1H, *N*4am-CH*H*), 1.51–1.38 (m, 5H, 2 × *N*3m-C_2_*H*_2_, *N*4am-CH*H*), 1.33–1.20 (m, 6H, 2 × *N*3m-C_3_*H*_2_, *N*4am-N*H*_2_), 0.90 (t, *J* = 7.3 Hz, 3H, *N*3m-C*H*_3_), 0.86 (t, *J* = 7.5 Hz, 3H, *N*3m-C*H*_3_). – **^13^C-NMR** (125 MHz, acetonitrile‑*d_3_*, ppm): δ = 172.5 (C_q_, *C*ON), 171.2 (C_q_, *C*ON), 170.6 (C_q_, *C*ON), 170.2 (C_q_, *C*ON), 170.1 (C_q_, *C*ON), 169.6 (C_q_, *C*ON), 139.8 (C_q_, *C*_ar_), 138.6 (C_q_, *C*_ar_), 138.0 (C_q_, *C*_ar_), 130.5 (+, 2 × *C*_ar_H), 130.3 (+, 2 × *C*_ar_H), 129.5 (+, 2 × *C*_ar_H), 129.4 (+, 2 × *C*_ar_H), 129.0 (+, 2 × *C*_ar_H), 128.8 (+, 2 × *C*_ar_H), 128.2 (+, *C*_ar_H), 127.8 (+, 2 × *C*_ar_H), 57.3 (+, phe-*C*_α_H), 55.5 (+, phe-*C*_α_H), 51.2 (–, CO*C*H_2_N), 51.0 (–, CO*C*H_2_N), 49.7 (–, *N*3m-*C*_1_H_2_), 49.2 (–, CO*C*H_2_N), 49.0 (–, CO*C*H_2_N), 48.8 (–, *N*3m-*C*_1_H_2_), 47.2 (–, *N*1ph-*C*H_2_), 44.2 (–, *N*4am-*C*_1_H_2_), 40.2 (–, *N*4am-*C*_4_H_2_), 38.1 (–, phe-*C*H_2_), 37.2 (–, phe-*C*H_2_), 31.2 (–, *N*3m-*C*_2_H_2_), 30.5 (–, *N*3m-*C*_2_H_2_), 24.2 (–, *N*4am-*C*H_2_), 23.1 (–, *N*4am-*C*H_2_), 20.7 (–, *N*3m-*C*_3_H_2_), 20.5 (–, *N*3m-*C*_3_H_2_), 14.1 (+, *N*3m-*C*H_3_), 14.0 (+, *N*3m-*C*H_3_).– **Analytical HPLC** (5–95% acetonitrile + 0.1% TFA in 30 min, detection at 218 nm): t_Ret_ = 17.3 min (>99%). – **MS**(m/z, MALDI-TOF): 796 [M+H]^+^, 818 [M+Na]^+^, 834 [M+K]^+^.

*cyclo*-(l‑Phe-*N*3m-*N*3m-*N*1ph-*N*4am(Rhod)-l‑Phe) (24-Rhod)

Macrocycle **24** (5.00 mg, 6.28 µmol, 1.00 equiv.) was conjugated to rhodamine B following **GP8**. After purification *via* preparative reversed-phase HPLC (5–95% acetonitrile in water with 0.1% TFA in 35 min), the title compound was isolated as a pink solid (1.10 mg, 0.90 µmol, 14%).

**Analytical HPLC** (5–95% acetonitrile + 0.1% TFA in 30 min, detection at 218 nm): t_Ret_ = 20.5 min (>99%). – **MS**(m/z, MALDI-TOF): 1221 [M+H]^+^, 1243 [M+Na]^+^.

**Table S1:** Analytical HPLC traces of macrocycles **15-24** and their conjugates **15-Rhod-24-Rhod**.

| 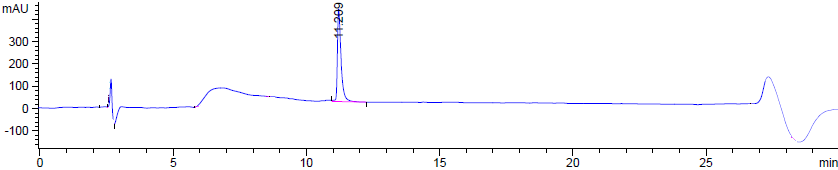**15** | 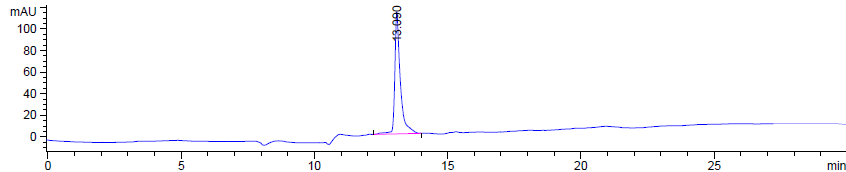  **15-Rhod** |
| --- | --- |
| 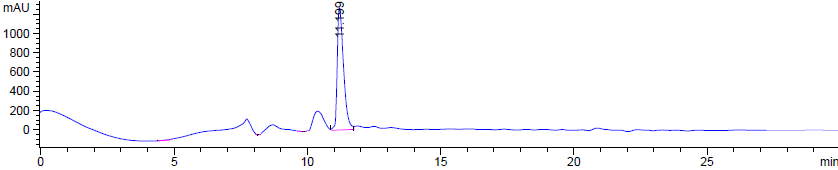**16** | 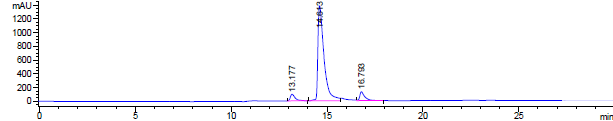  **16-Rhod** |
| 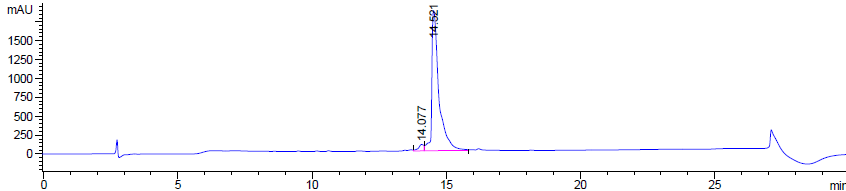  **17** | 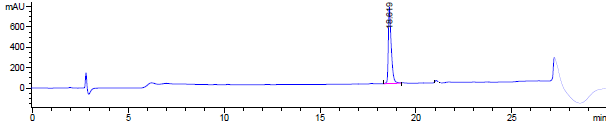**17-Rhod** |
| 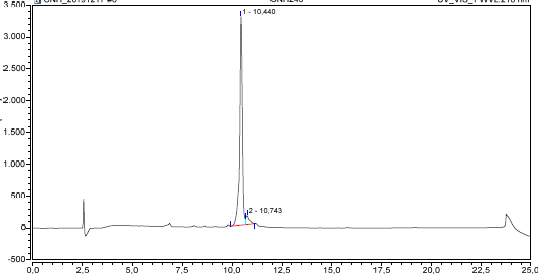  **18** | 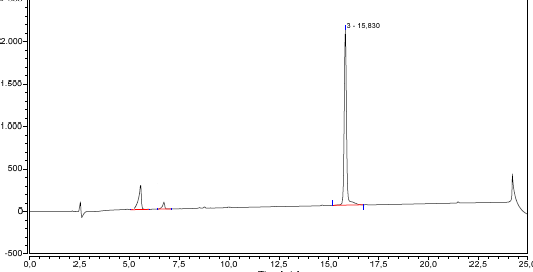**18-Rhod** |
| 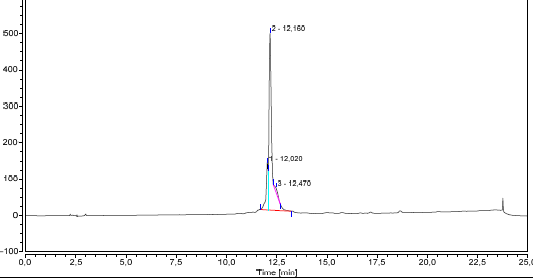  **19** | 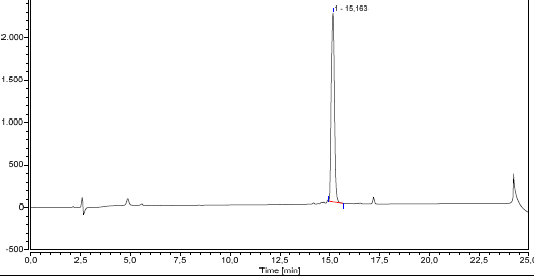  **19-Rhod** |
| 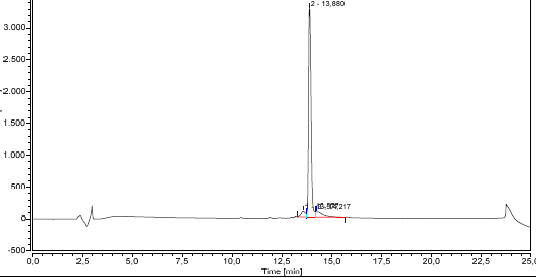  **20** | 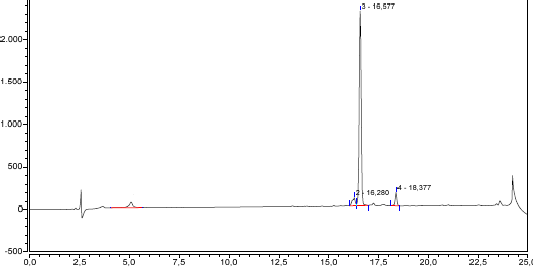**20-Rhod** |
| 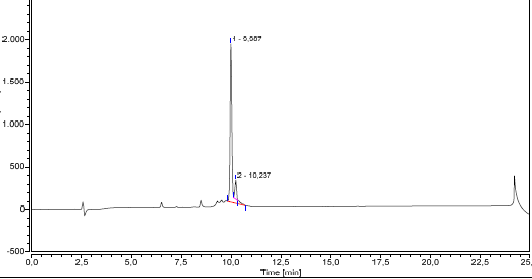  **21** | 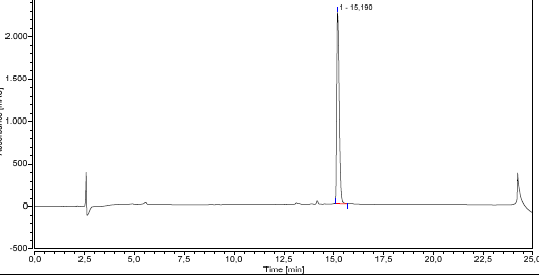  **21-Rhod** |
| 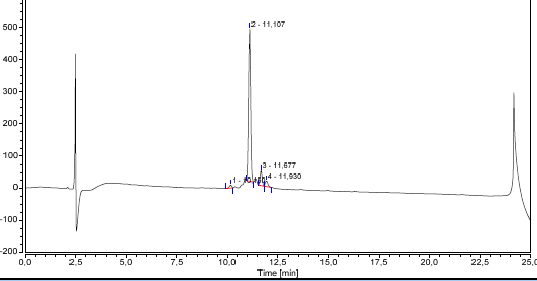  **22** | 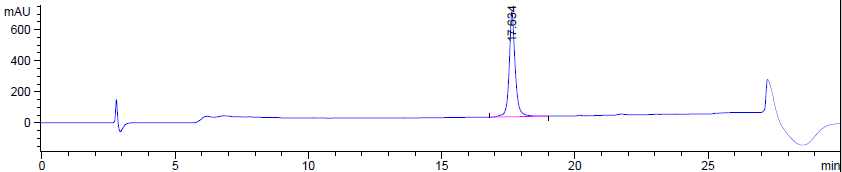  **22-Rhod** |
| 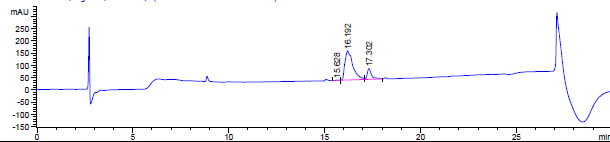  **23** | 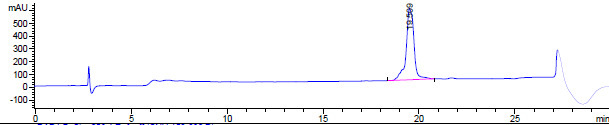  **23-Rhod** |
| 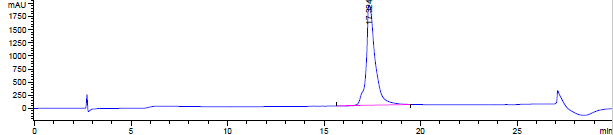  **24** | 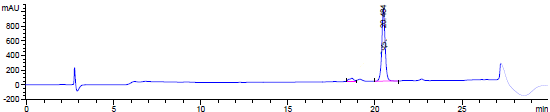  **24-Rhod** |

T**able S2:** MALDI-spectra of macrocycles **15-24** and their conjugates **15-Rhod-24-Rhod**.

| 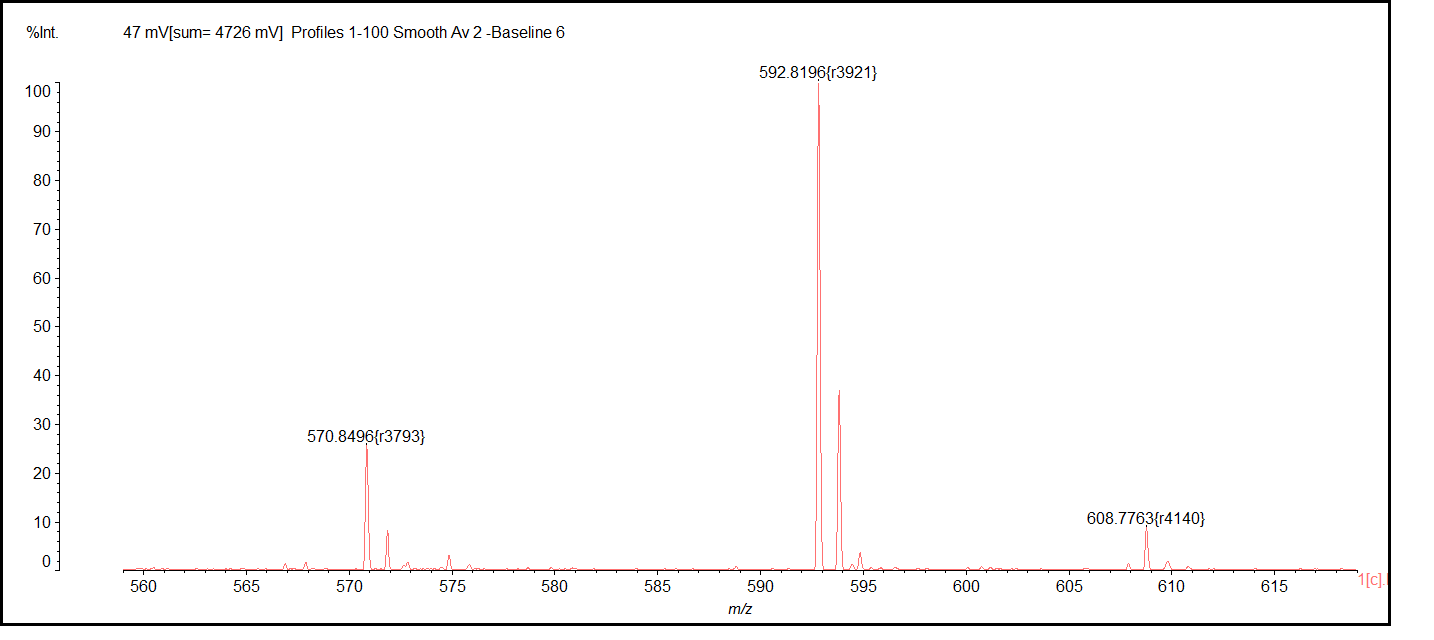**15** | 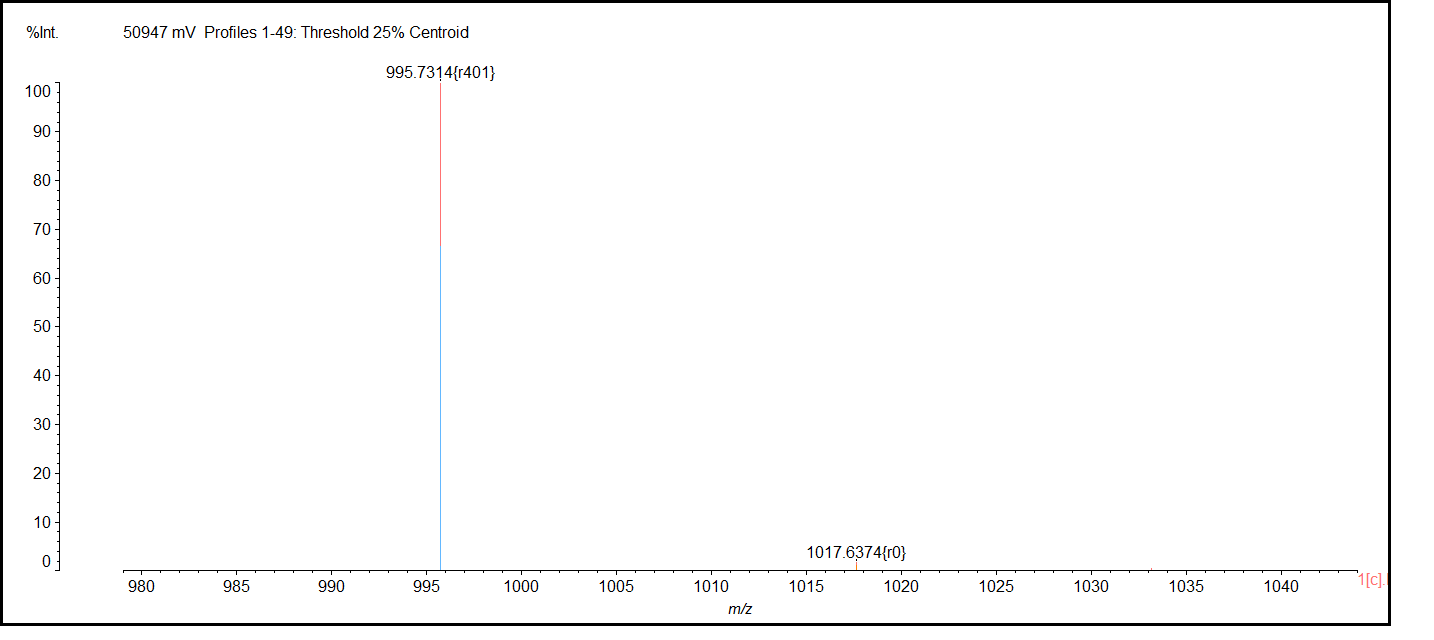**15-Rhod** |
| --- | --- |
| 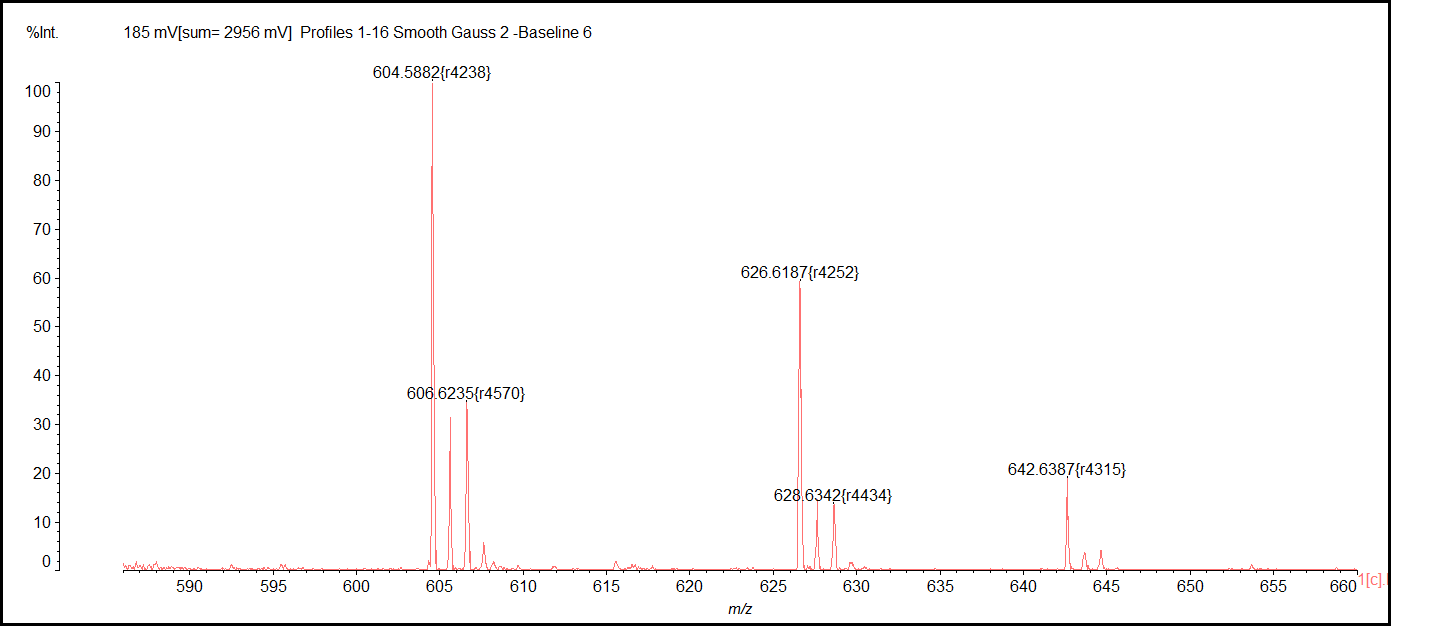**16** | 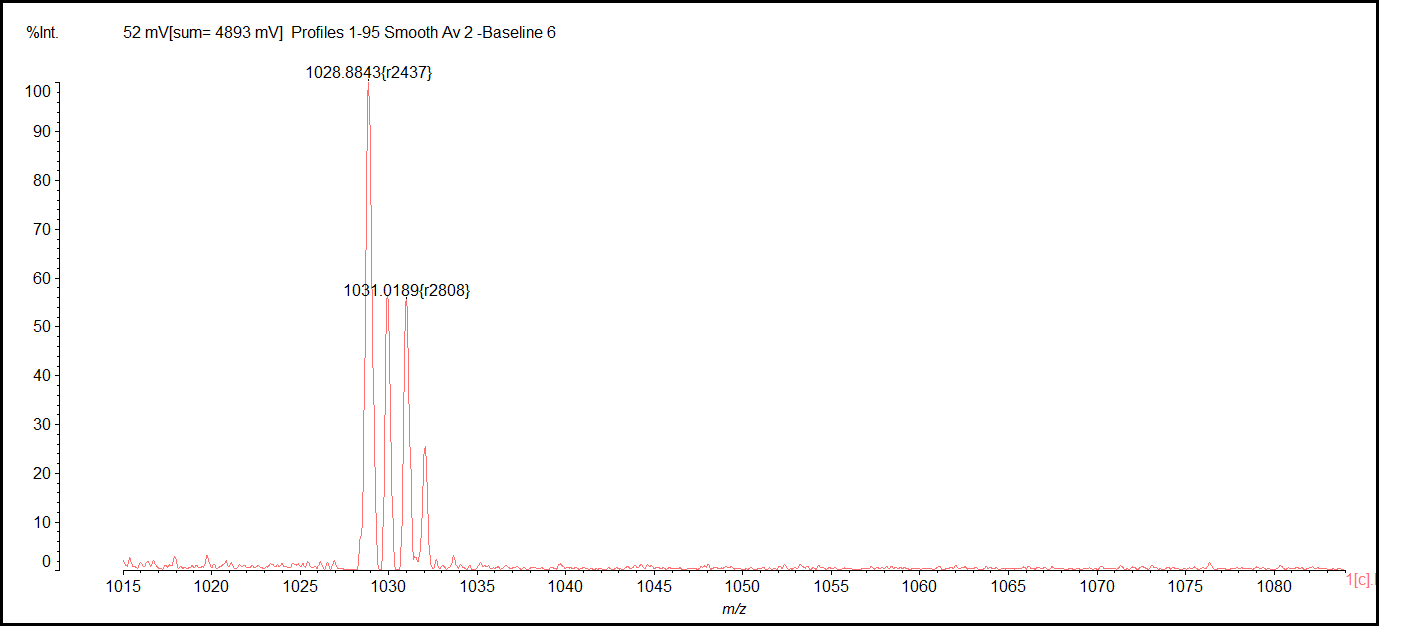**16-Rhod** |
| 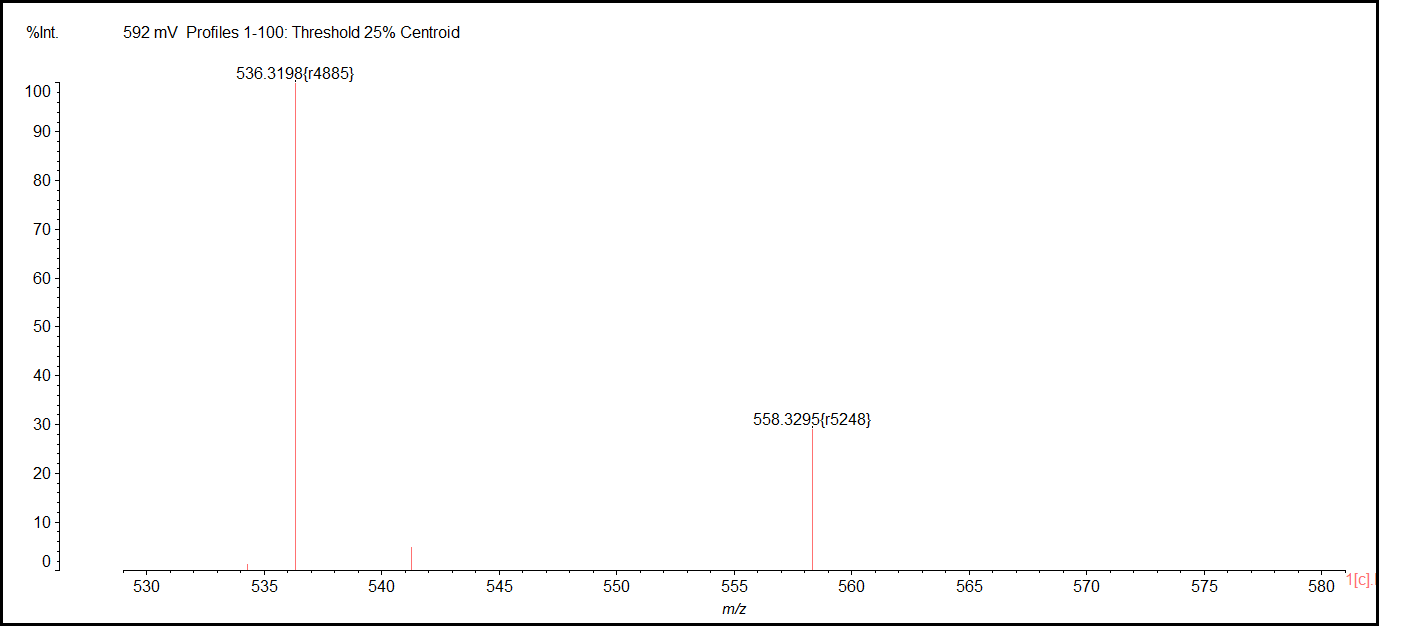**17** | 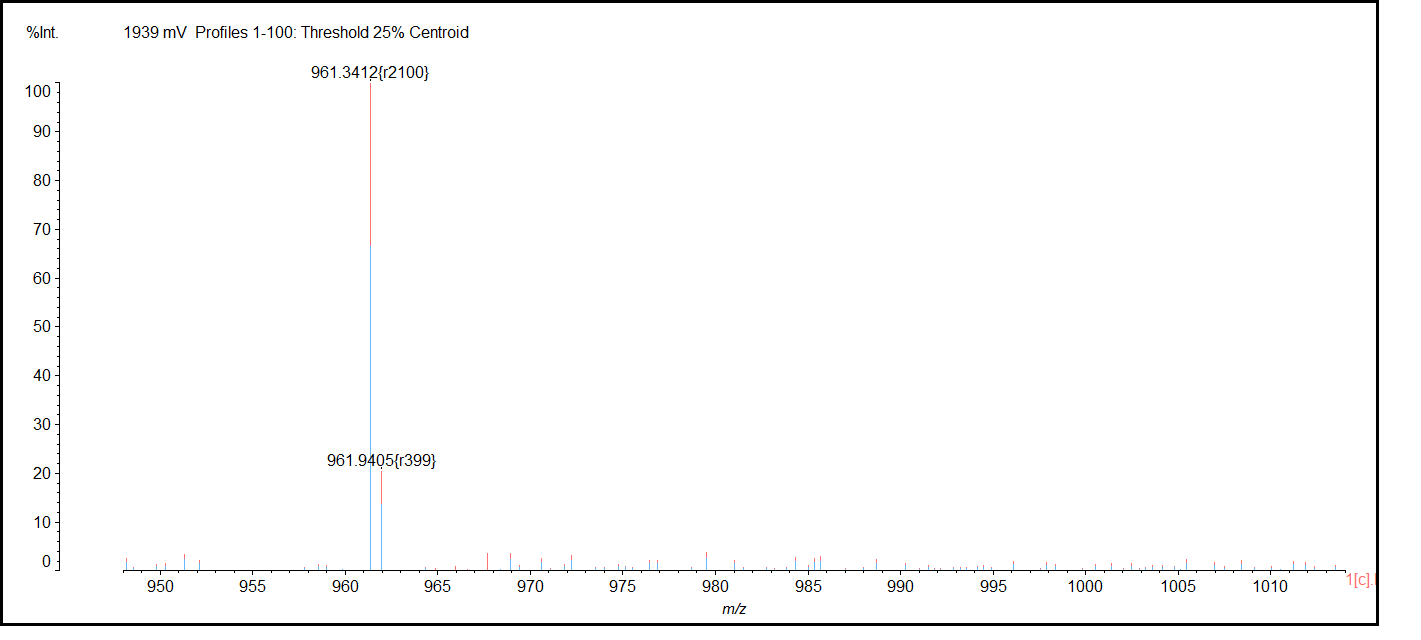**17-Rhod** |
| 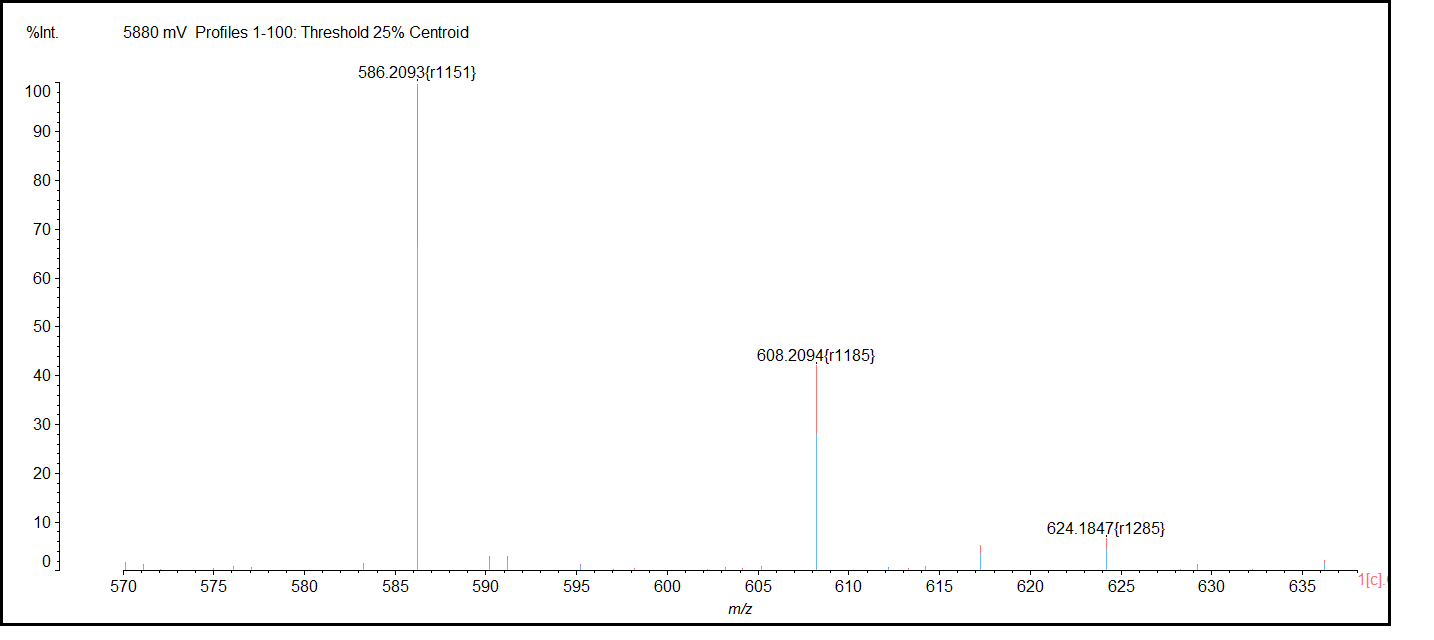**18** | 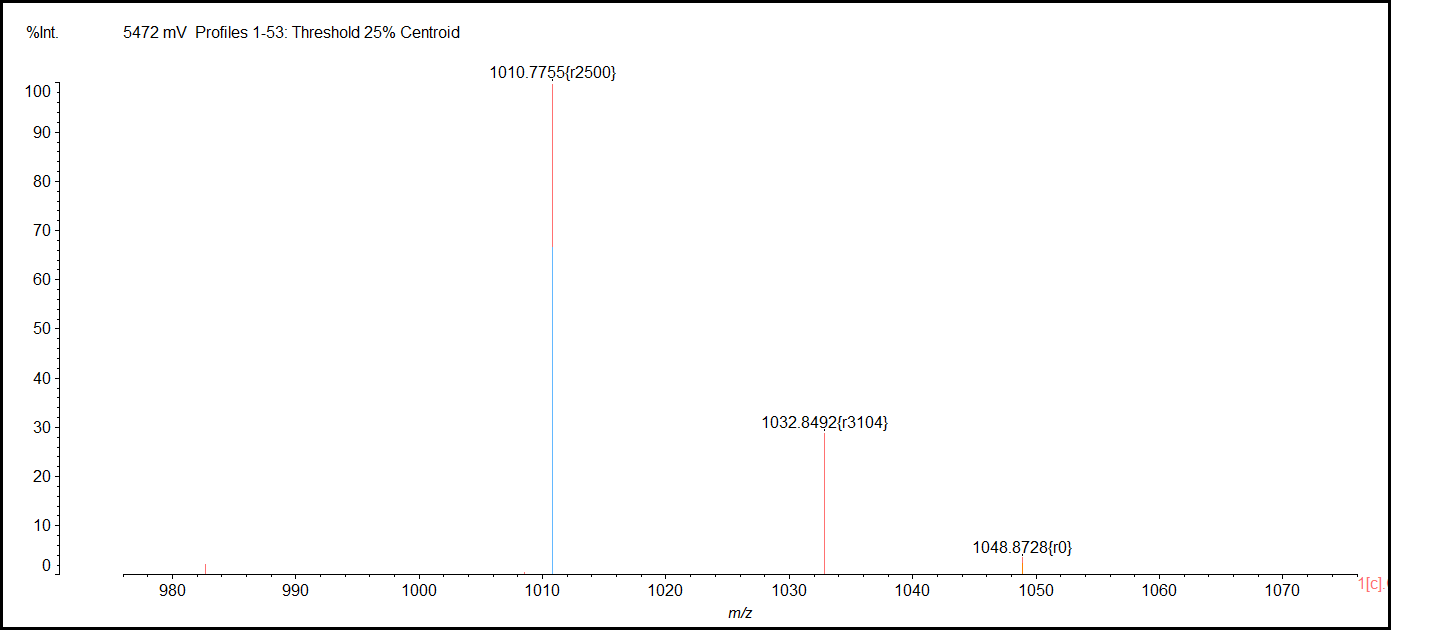**18-Rhod** |
| 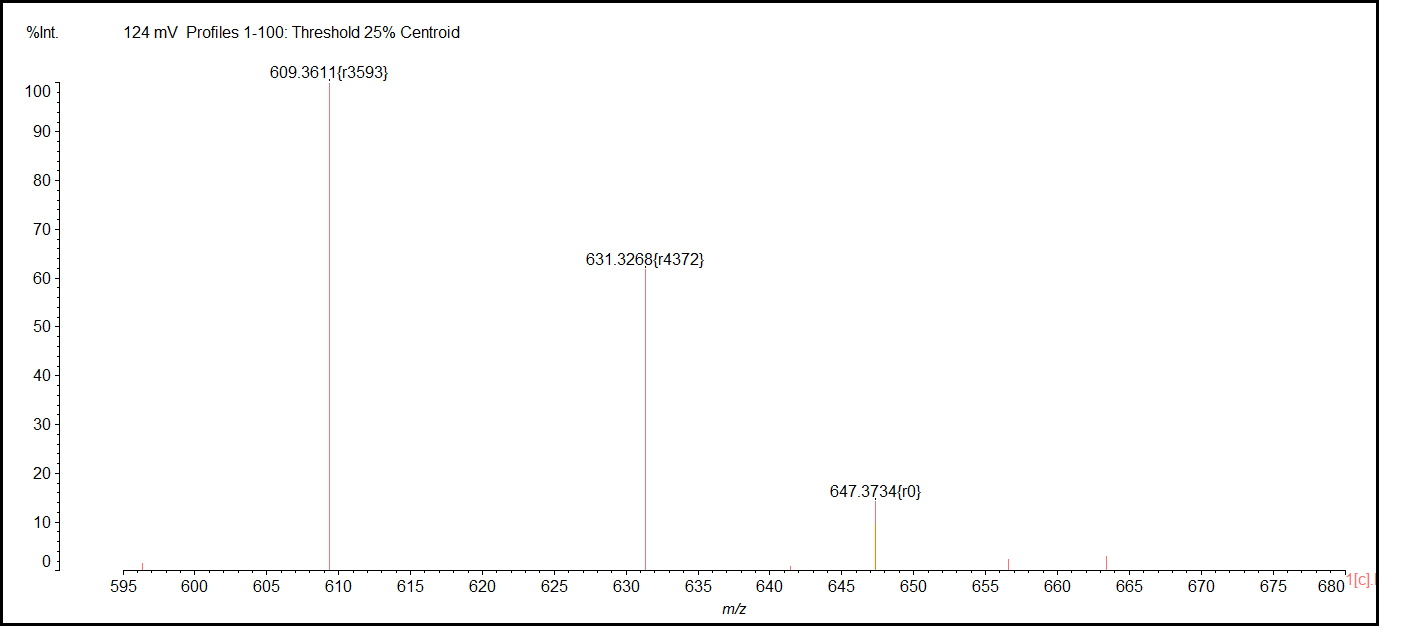**19** | 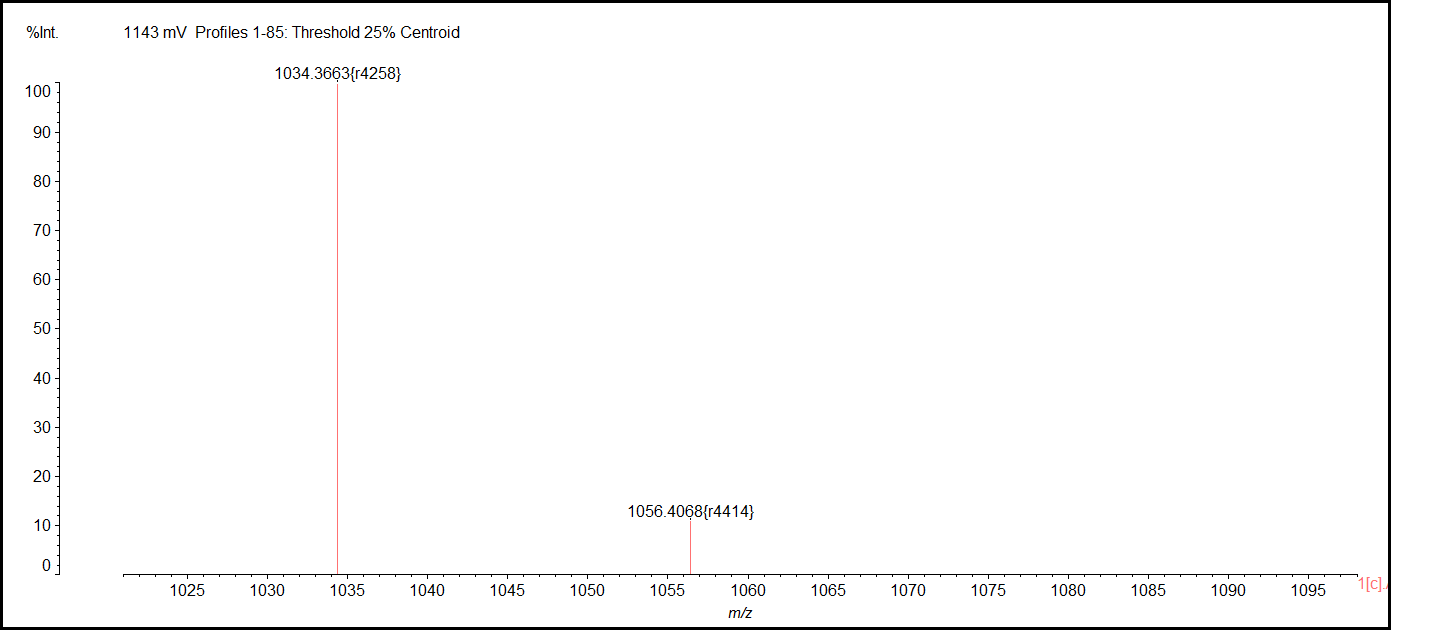**19-Rhod** |
| 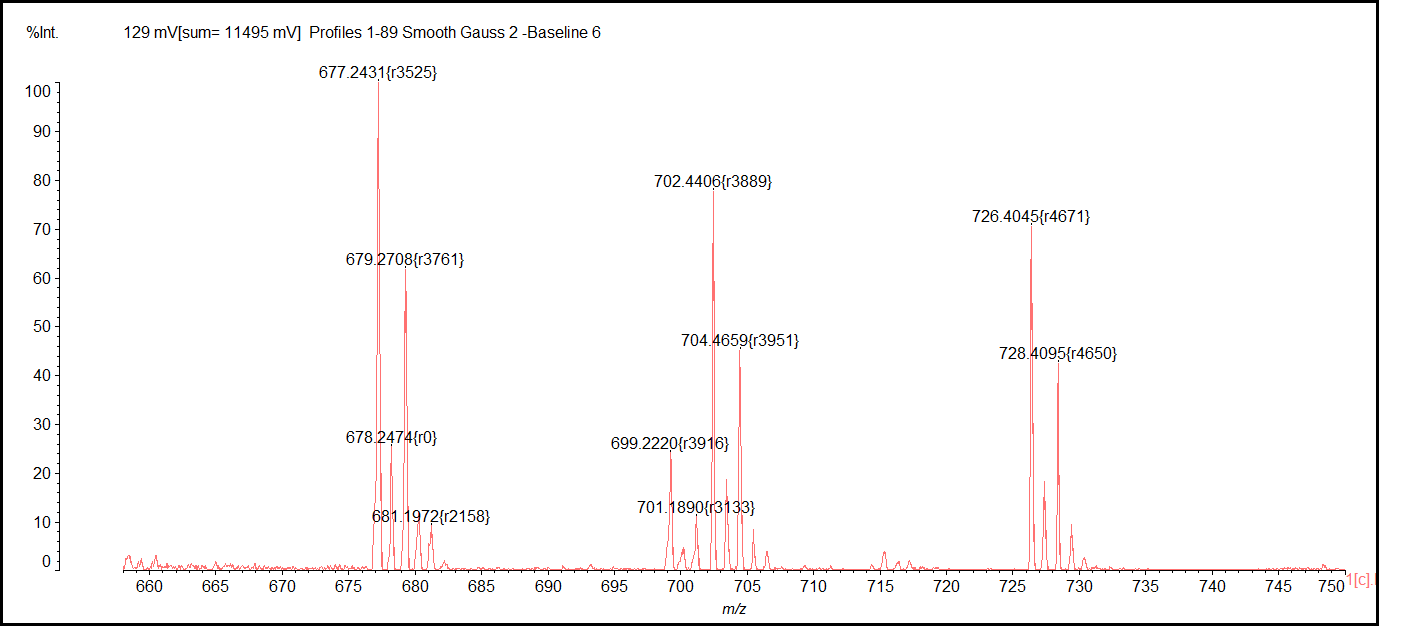**20** | 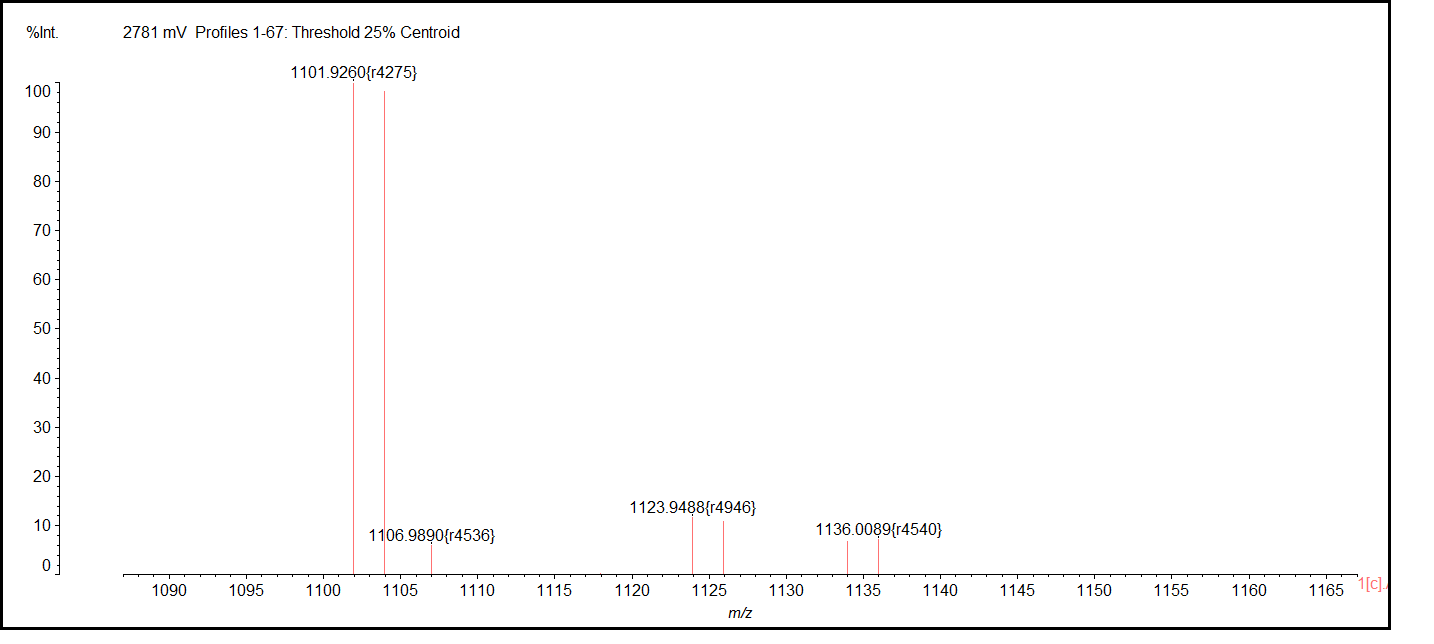**20-Rhod** |
| 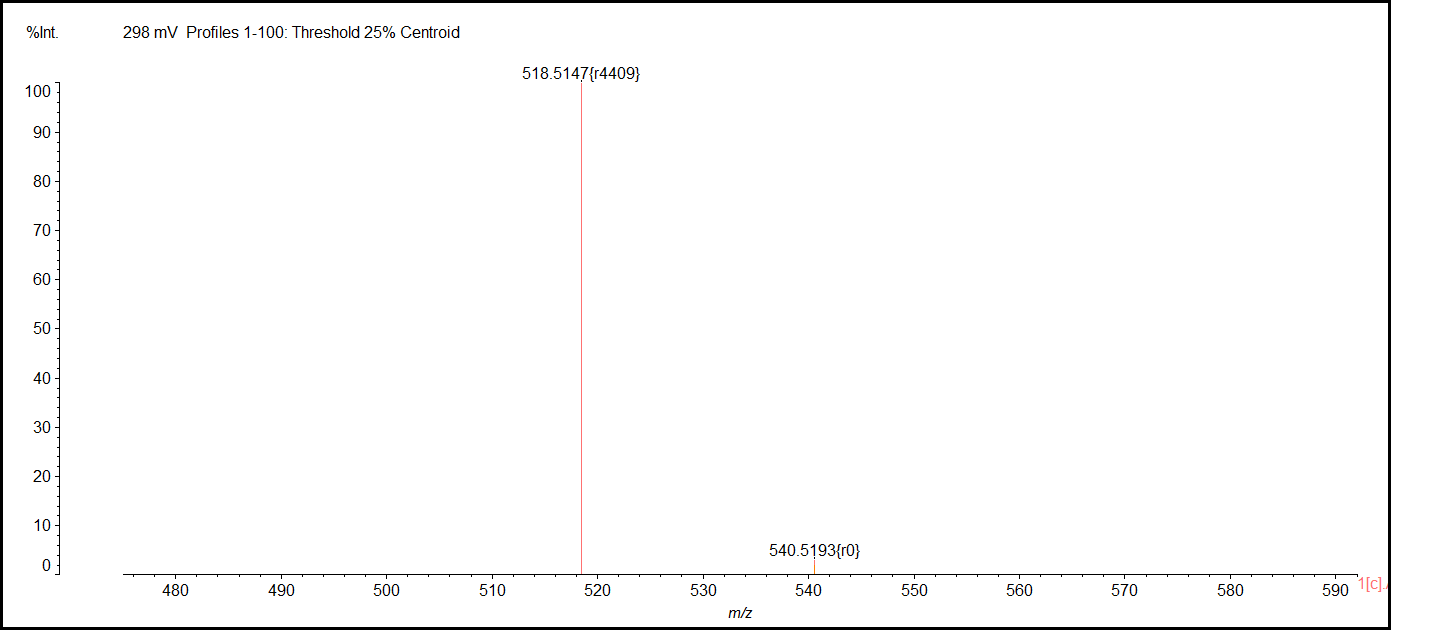**21** | 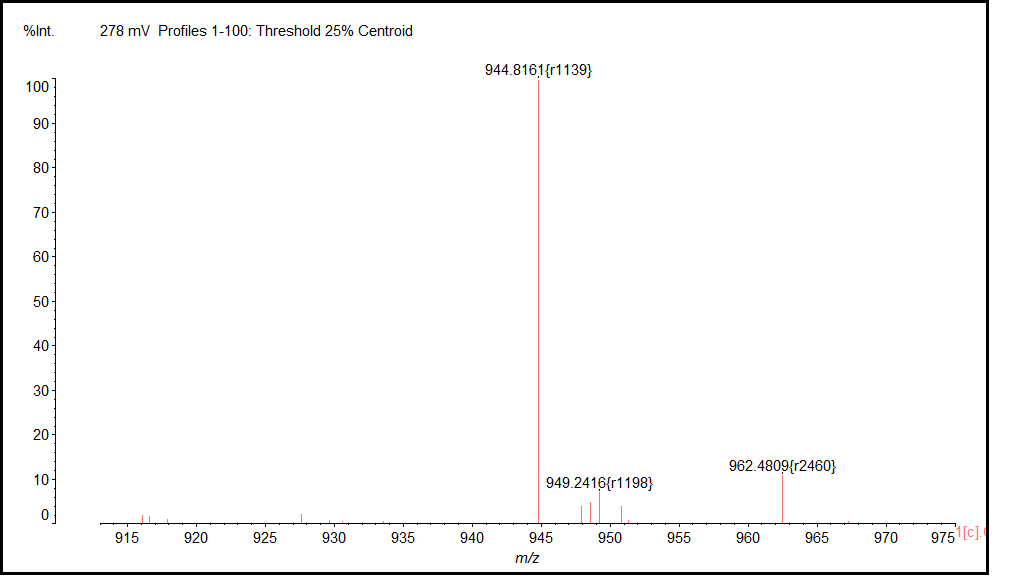  **21-Rhod** |
| 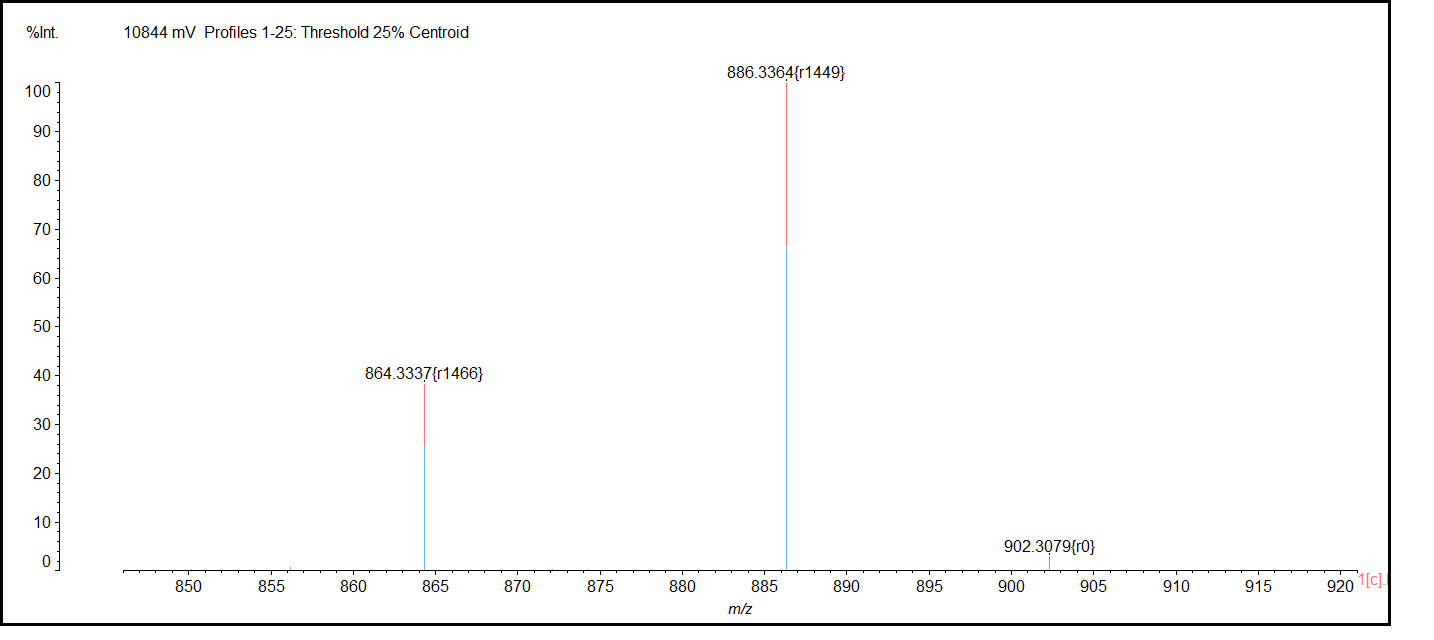**22** | 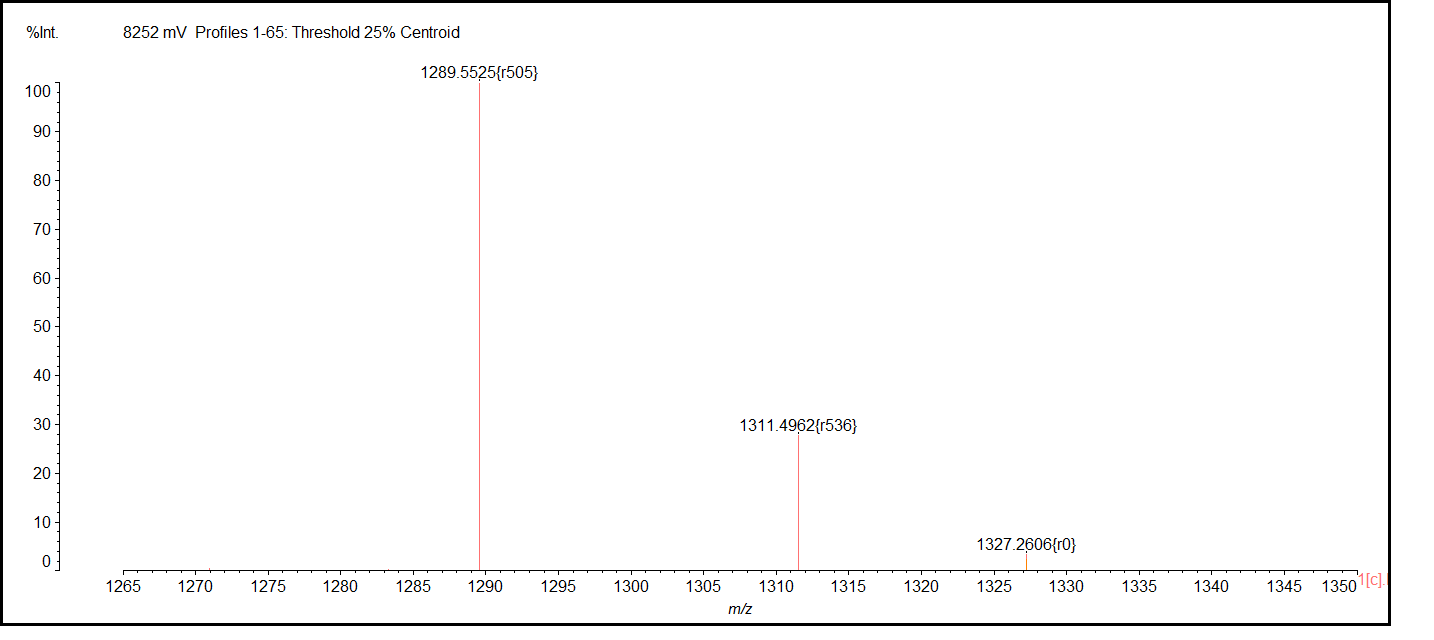**22-Rhod** |
| 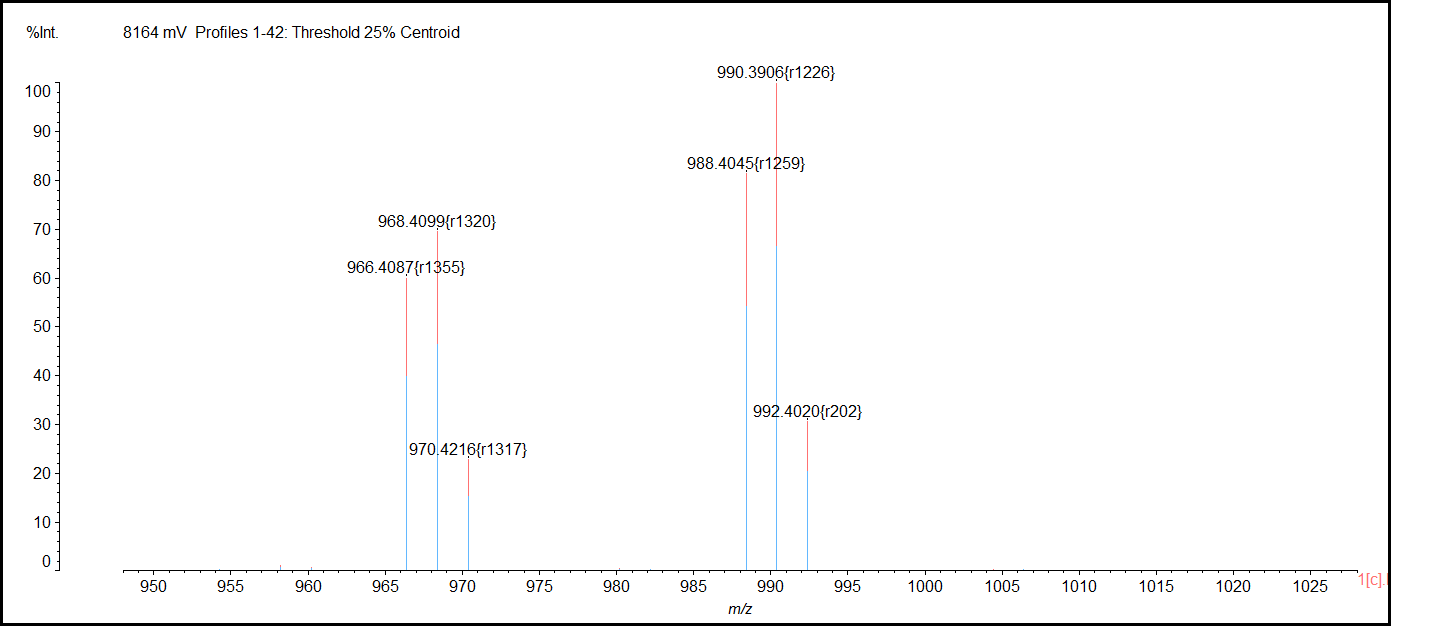**23** | 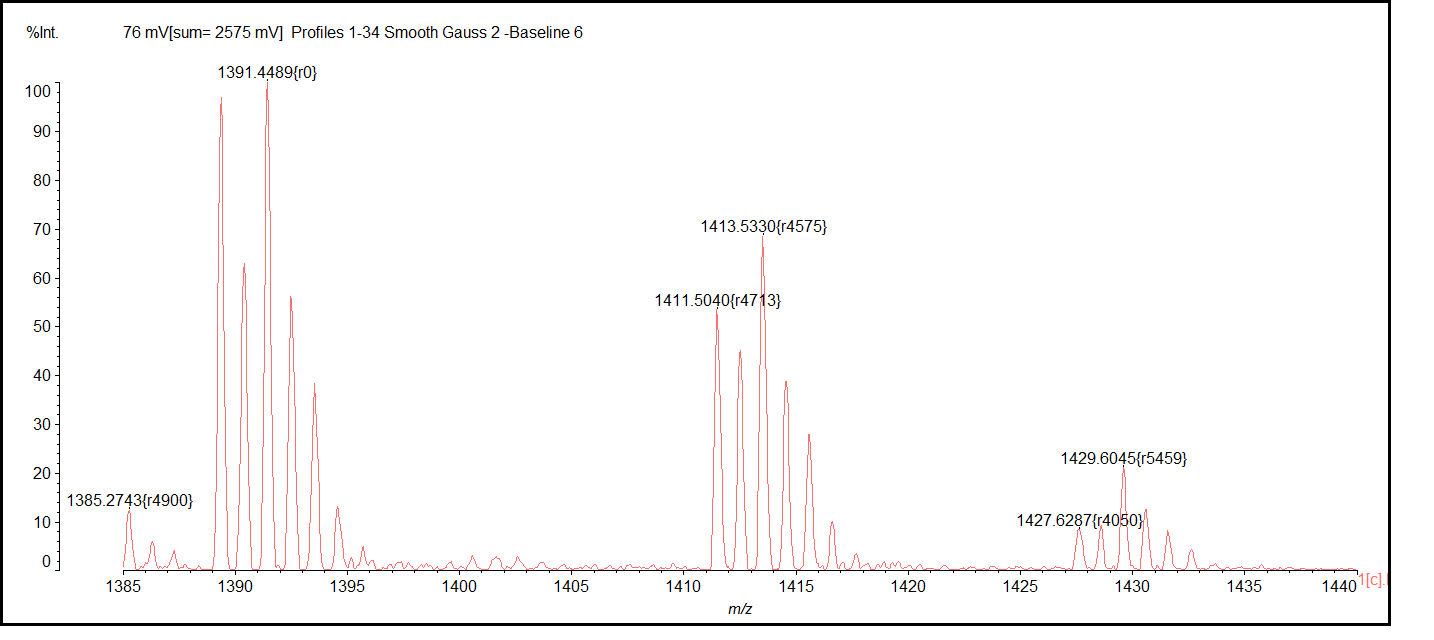**23-Rhod** |
| 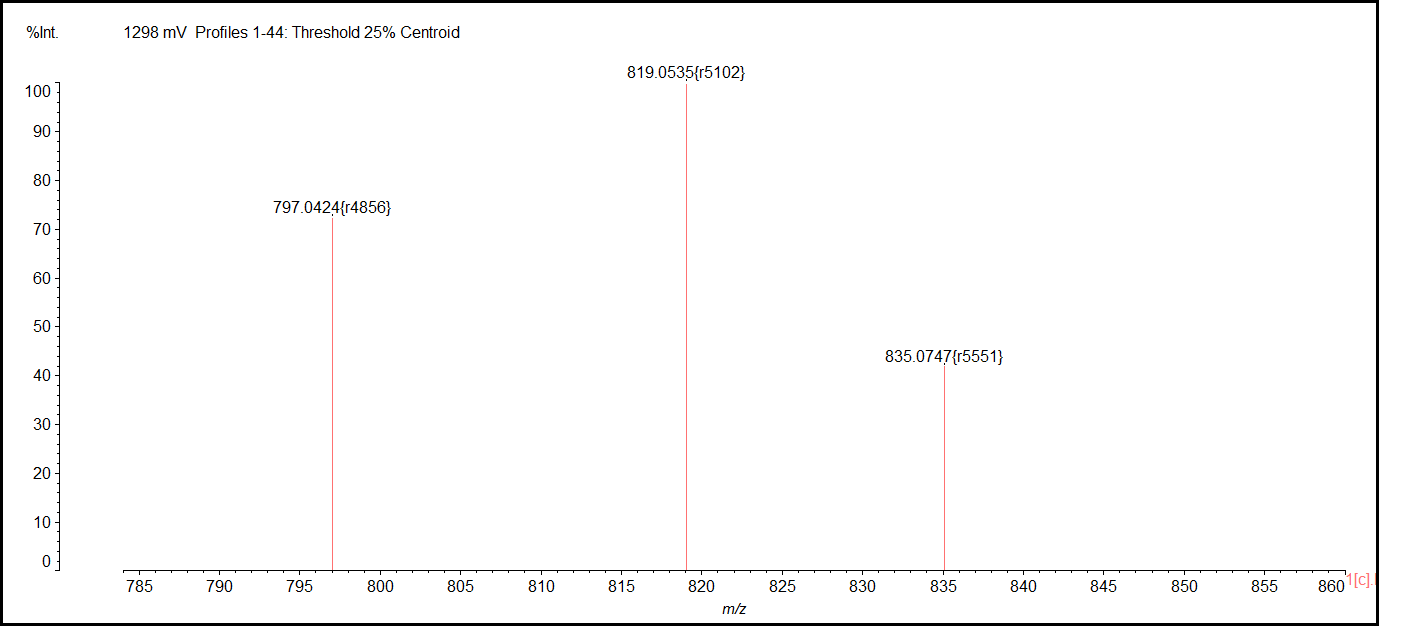**24** | 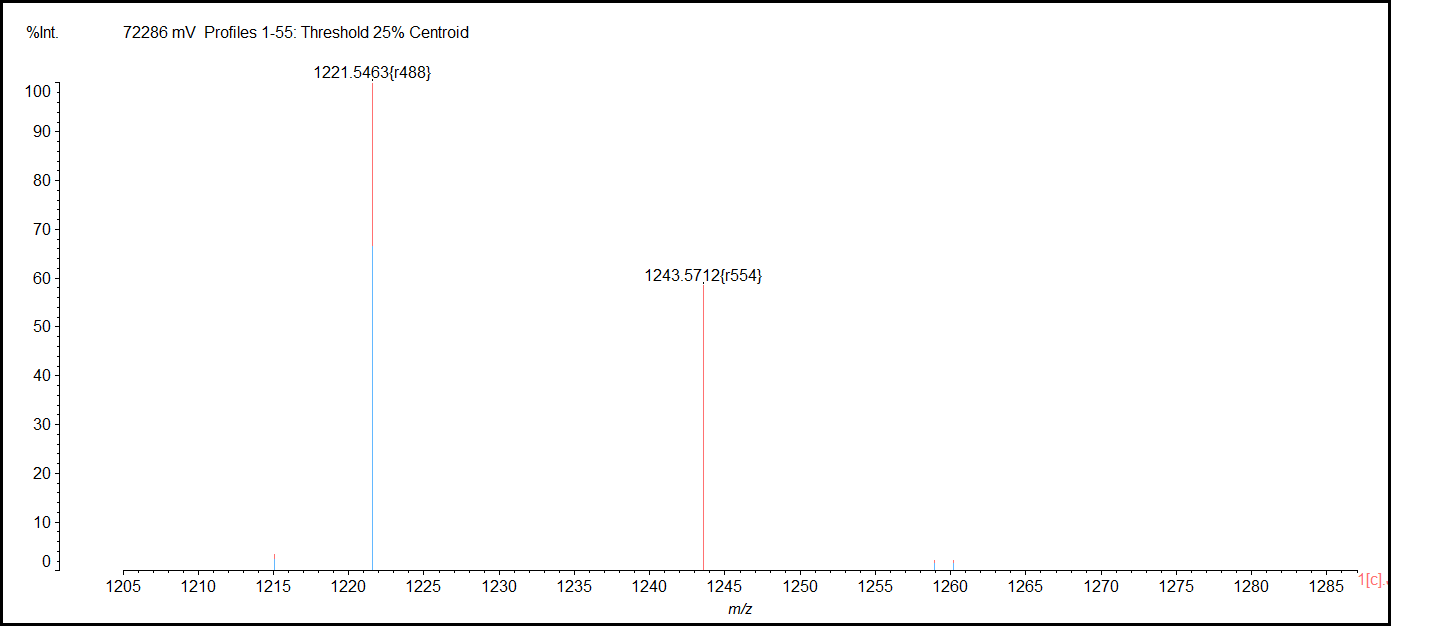**24-Rhod** |

**Table S3**: NMR-spectra of macrocycles **15-24**.

| ***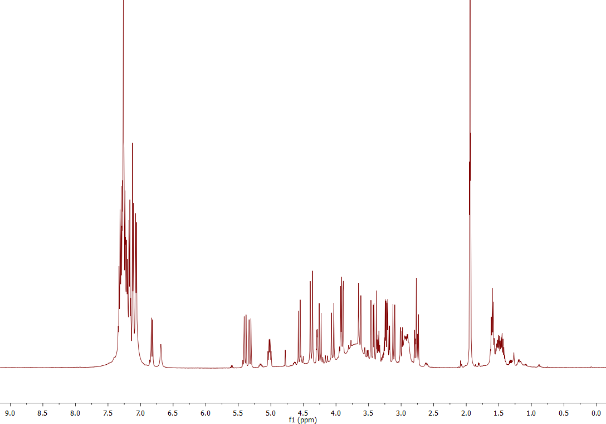***  **15** | ***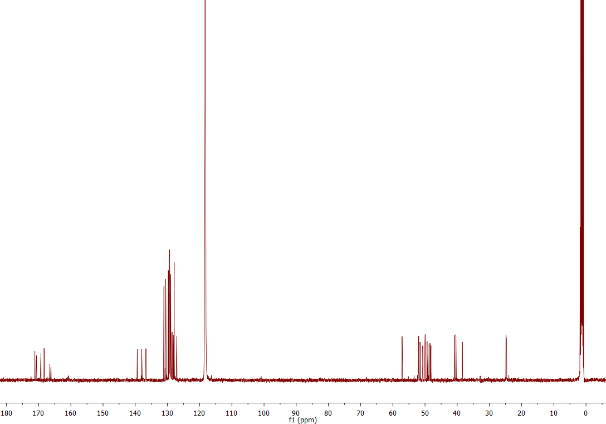***  **15** |
| --- | --- |
| 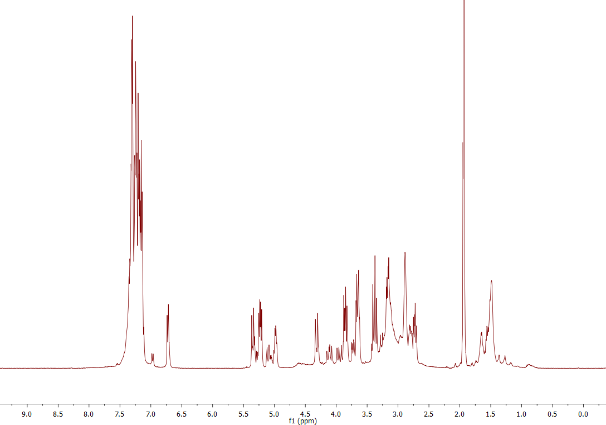  **16** | 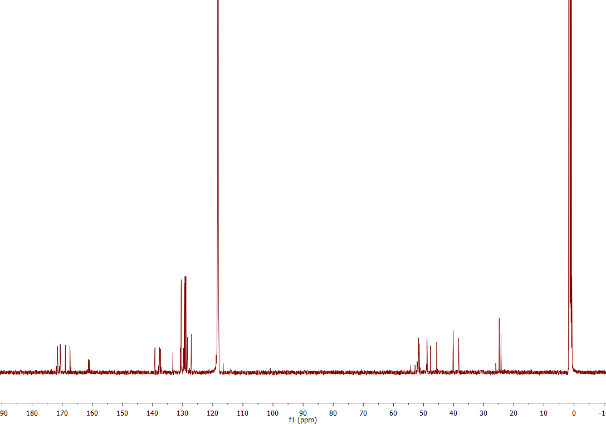  **16** |
| 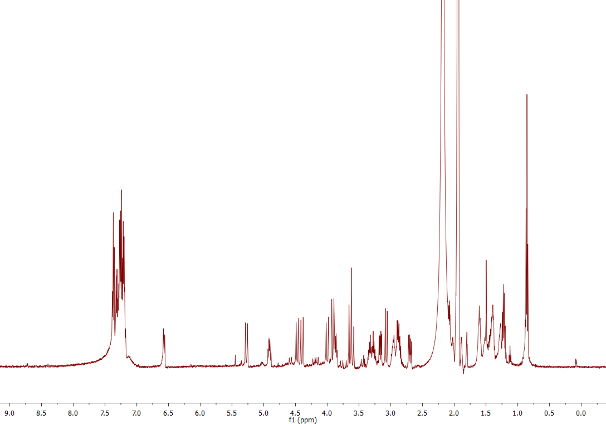  **17** | 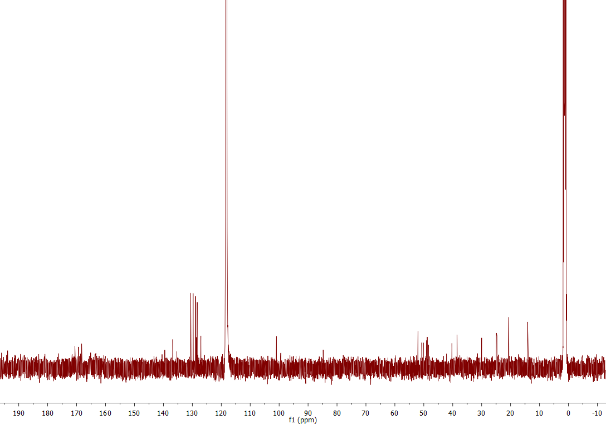  **17** |
| 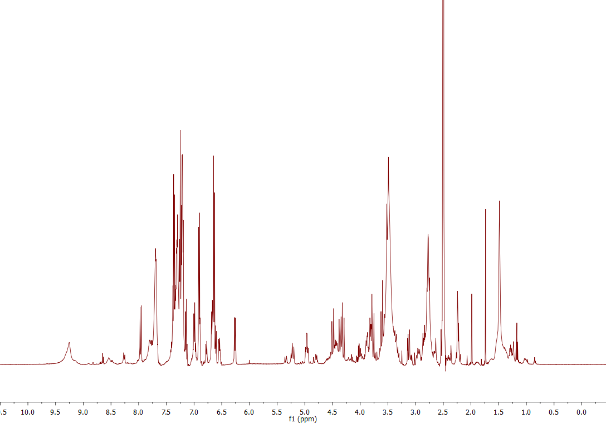  **18** | 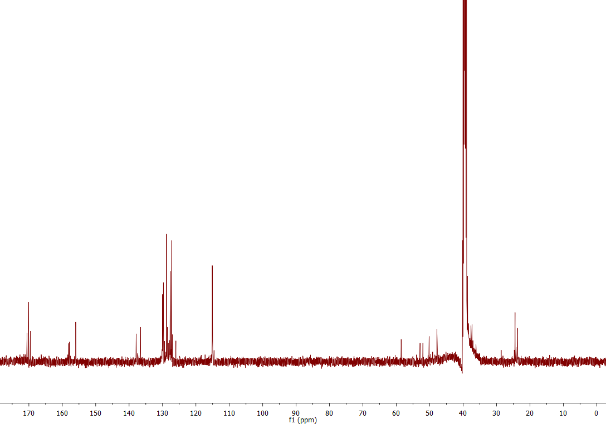  **18** |
| 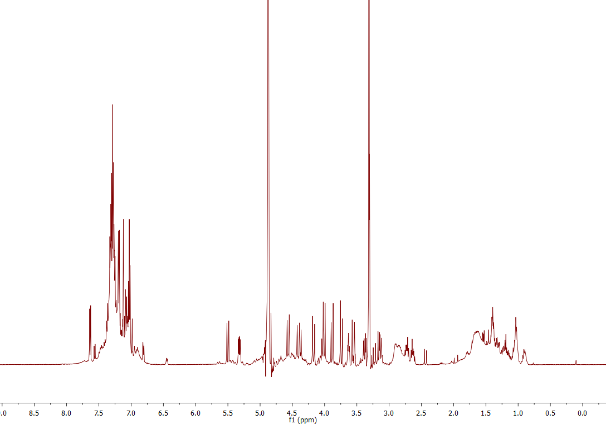  **19** | 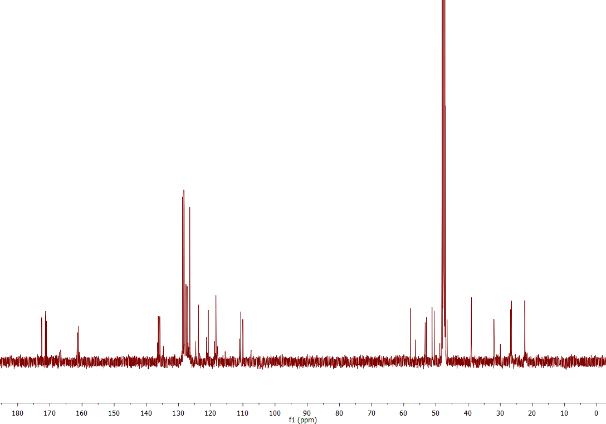  **19** |
| 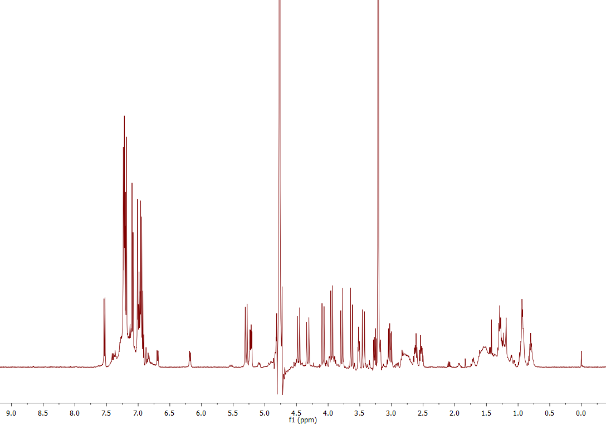  **20** | 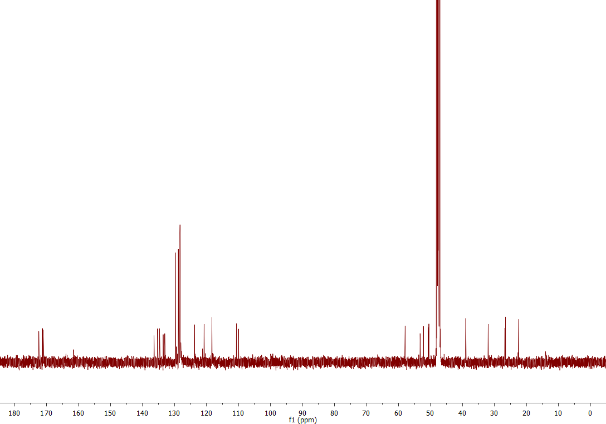  **20** |
| 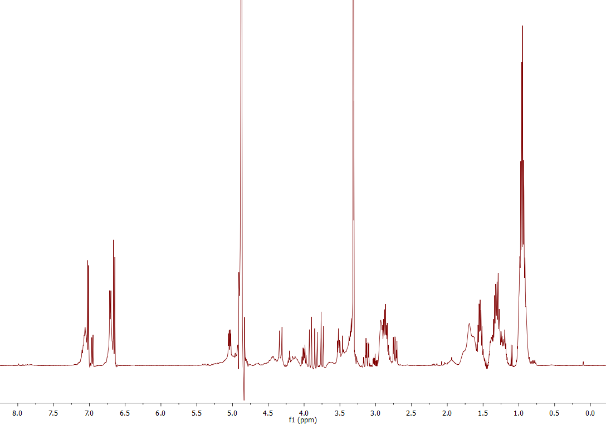  **21** | 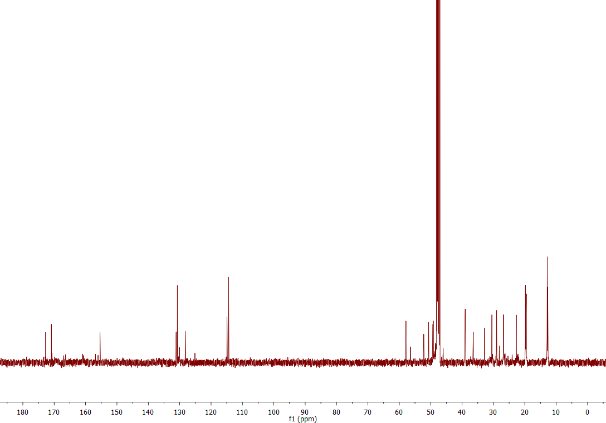  **21** |
| 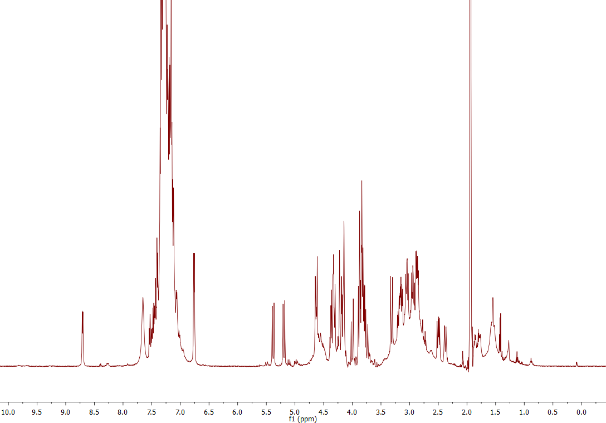  **22** | 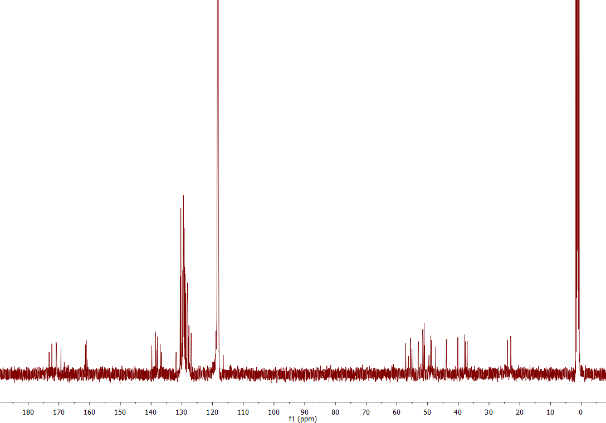  **22** |
| 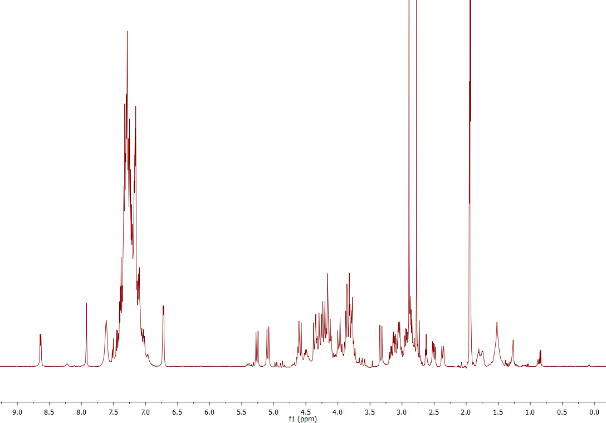  **23** | 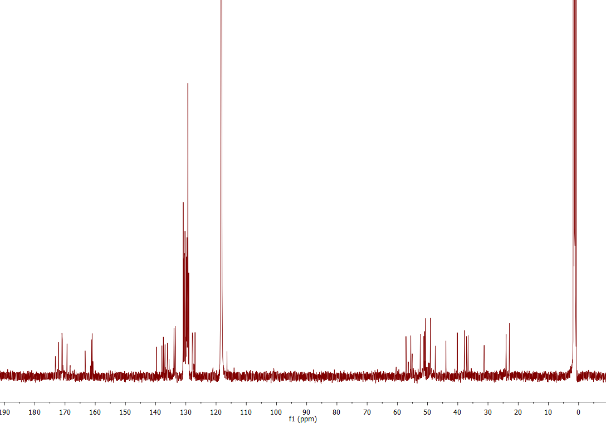  **23** |
| 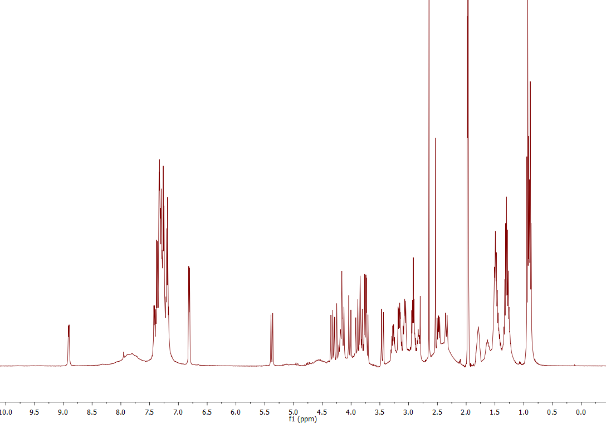  **24** | 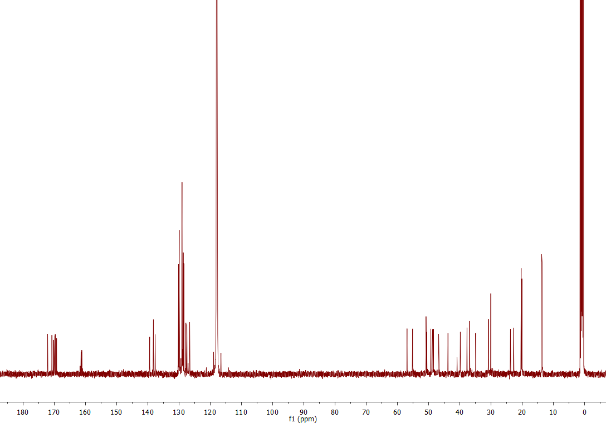  **24** |

**References**

1. Mosmann T. Rapid colorimetric assay for cellular growth and survival: application to proliferation and cytotoxicity assays. J Immunol Methods. 1983;65:55-63.

2. Merrifield RB. Solid phase peptide synthesis. I. The synthesis of a tetrapeptide. Journal of the American Chemical Society. 1963;85(14):2149-54.

3. Simon RJ, Kania RS, Zuckermann RN, Huebner VD, Jewell DA, Banville S, et al. Peptoids: a modular approach to drug discovery. Proc Natl Acad Sci U S A. 1992;89(20):9367-71.

4. Aldrich JV, Kulkarni SS, Senadheera SN, Ross NC, Reilley KJ, Eans SO, et al. Unexpected Opioid Activity Profiles of Analogues of the Novel Peptide Kappa Opioid Receptor Ligand CJ‐15,208. ChemMedChem. 2011;6(9):1739-45.

5. Schröder T, Schmitz K, Niemeier N, Balaban TS, Krug HF, Schepers U, et al. Solid-phase synthesis, bioconjugation, and toxicology of novel cationic oligopeptoids for cellular drug delivery. Bioconjugate chemistry. 2007;18(2):342-54.
